# Supplementary figures and images for: Were the socio-economic determinants of municipalities relevant to the increment of COVID-19 related deaths in Brazil in 2020?
Source: PLoS One. 2022 Apr 28;17(4):e0266109. doi: 10.1371/journal.pone.0266109 (PMC9049518; doi:10.1371/journal.pone.0266109)

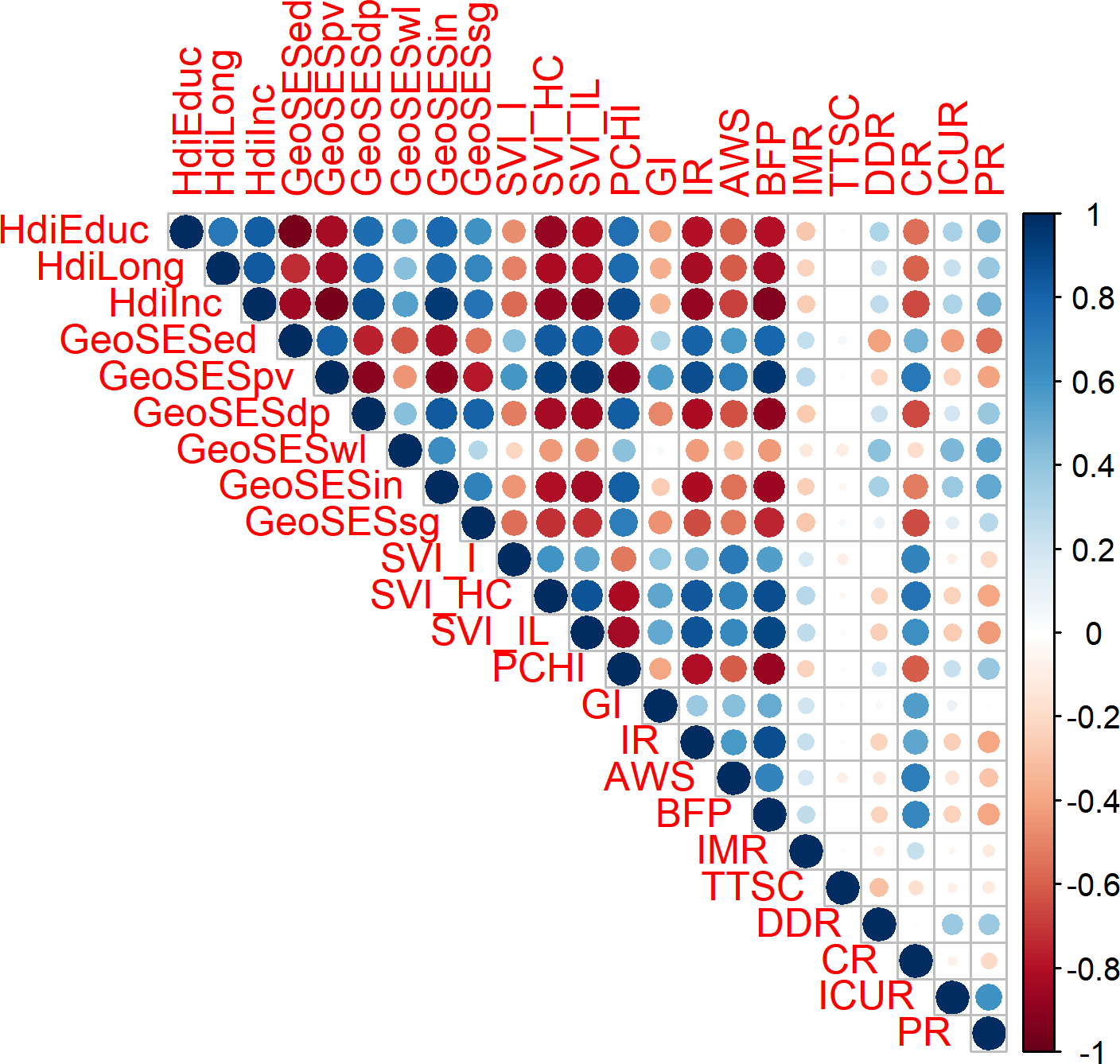

Supplement: S1 Fig — Dark and light, red and blue circles represent the intensity of correlations. (TIF) [file pone.0266109.s001.tif]
