# Supplementary material for: Were the socio-economic determinants of municipalities relevant to the increment of COVID-19 related deaths in Brazil in 2020?
Source: PLoS One. 2022 Apr 28;17(4):e0266109. doi: 10.1371/journal.pone.0266109 (PMC9049518; doi:10.1371/journal.pone.0266109)
Supplement: S1 Table — Brazil, 2020. (PDF) [file pone.0266109.s002.pdf]

Suppl. Table 1. Robustness Check of Adjusted Log-linear Models to Estimate Association Between COVID-19 Mortality Rate (CMR, SMR and SMRT) and Socioeconomic Status in Brazilian Municipalities. Brazil, 2020.

	Dependent variable:											
	CMR				SMR				SMRT			
	(1)	(2)	(3)	(4)	(5)	(6)	(7)	(8)	(9)	(10)	(11)	(12)
SVI Human Capital	−24.24**				−21.68				−0.20			
	[−43.79;−4.69]				[−66.62;23.25]				[−0.57;0.16]			
SVI Labor and Income	−57.83***				−253.28***				−1.13***			
	[−74.96;−40.69]				[−292.54;−214.02]				[−1.45;−0.81]			
SVI Urban Infrastructure	−8.12*				−6.01				−0.18**			
	[−17.53;1.29]				[−27.53;15.52]				[−0.36;−0.01]			
HDI Education		31.37**				90.16***				0.77***		
		[6.57;56.18]				[33.64;146.67]				[0.31;1.23]		
HDI Health		−14.95				−58.91				−0.92*		
		[−64.27;34.38]				[−172.56;54.74]				[−1.84;0.01]		
HDI Income		183.14***				562.36***				2.46***		
		[144.81;221.46]				[474.45;650.28]				[1.74;3.18]		
GeoSES Education			−0.40***				−1.18***				−0.004	
			[−0.69;−0.12]				[−1.82;−0.54]				[−0.01;0.001]	
GeoSES Poverty			−0.86***				−2.37***				−0.01***	
			[−1.07;−0.66]				[−2.83;−1.90]				[−0.02;−0.01]	
GeoSES Deprivation			−0.34***				−0.33				0.001	
			[−0.57;−0.11]				[−0.85;0.19]				[−0.003;0.01]	
GeoSES Wealth			0.31				−4.15*				−0.09***	
			[−1.82;2.44]				[−8.99;0.68]				[−0.13;−0.05]	
GeoSES Income			−0.001				0.001				0.0000	
			[−0.01;0.003]				[−0.01;0.01]				[−0.0001;0.0001]	
GeoSES Segregation			−19.55				−131.65***				−0.93**	
			[−62.74;23.63]				[−229.88;−33.41]				[−1.73;−0.12]	
Household Per Capita Income				0.03***				0.09***				0.0003***
				[0.02;0.05]				[0.06;0.12]				[0.0001;0.0005]
Gini Index				−31.50***				−81.75***				−1.17***
				[−52.52;−10.47]				[−130.65;−32.85]				[−1.57;−0.77]
Illiterate Rate				−0.24				−1.87***				−0.02***
				[−0.53;0.05]				[−2.53;−1.21]				[−0.02;−0.01]
Absence of Water and Sanitation				−0.44***				−1.15***				−0.01***
				[−0.59;−0.29]				[−1.48;−0.82]				[−0.01;−0.01]
BFP Recipients				−0.12				−0.38**				0.001
				[−0.28;0.04]				[−0.74;−0.02]				[−0.002;0.003]
Child Mortality Rate				−0.04				−0.03				−0.001
				[−0.19;0.10]				[−0.35;0.30]				[−0.004;0.002]
Crowding Rate	0.17*	0.29***	0.35***	0.25***	2.57***	3.04***	3.16***	2.91***	0.01***	0.01***	0.01***	0.01***
	[−0.02;0.36]	[0.12;0.46]	[0.16;0.53]	[0.07;0.42]	[2.14;3.00]	[2.64;3.43]	[2.73;3.59]	[2.52;3.31]	[0.004;0.01]	[0.01;0.01]	[0.01;0.01]	[0.01;0.01]
Density Rate	0.06***	0.05***	0.05***	0.06***	0.20***	0.17***	0.17***	0.19***	0.001***	0.001***	0.001***	0.001***
	[0.05;0.08]	[0.04;0.06]	[0.04;0.06]	[0.05;0.07]	[0.17;0.23]	[0.14;0.19]	[0.14;0.20]	[0.16;0.22]	[0.001;0.001]	[0.0005;0.001]	[0.001;0.001]	[0.001;0.001]
Travel Time	−0.005	−0.01	−0.01	−0.01	0.001	−0.01	−0.001	0.002	0.0001*	0.0001	0.0001*	0.0002**
	[−0.01;0.002]	[−0.01;0.001]	[−0.01;0.002]	[−0.01;0.002]	[−0.02;0.02]	[−0.02;0.01]	[−0.02;0.02]	[−0.01;0.02]	[−0.0000;0.0003]	[−0.0000;0.0002]	[−0.0000;0.0003]	[0.0000;0.0003]
State Capital	28.85***	20.36***	27.63***	25.61***	72.75***	45.78***	69.19***	70.12***	0.25**	0.10	0.28**	0.26**
	[16.28;41.42]	[8.08;32.63]	[14.90;40.36]	[13.38;37.83]	[42.18;103.31]	[15.68;75.89]	[38.06;100.32]	[39.79;100.44]	[0.002;0.49]	[−0.14;0.34]	[0.03;0.53]	[0.02;0.51]
ICU Hospital Beds Rate	−1.51***	−1.84***	−1.79***	−1.62***	−3.34***	−4.52***	−4.15***	−3.98***	−0.04***	−0.04***	−0.04***	−0.04***
	[−2.12;−0.91]	[−2.44;−1.24]	[−2.39;−1.18]	[−2.22;−1.02]	[−4.73;−1.94]	[−5.90;−3.13]	[−5.54;−2.76]	[−5.36;−2.59]	[−0.05;−0.03]	[−0.06;−0.03]	[−0.05;−0.03]	[−0.05;−0.03]
Physician Rate	3.83***	1.76*	3.13***	4.70***	5.92***	0.71	5.26**	7.56***	−0.02	−0.04**	0.002	0.002
	[1.93;5.72]	[−0.16;3.67]	[1.11;5.16]	[2.81;6.58]	[1.47;10.38]	[−3.81;5.22]	[0.51;10.02]	[3.08;12.03]	[−0.05;0.02]	[−0.08;−0.002]	[−0.04;0.04]	[−0.03;0.04]
Constant	97.23***	−62.17***	136.21***	75.57***	193.21***	−288.95***	277.25***	135.71***	3.24***	1.39***	3.47***	3.42***
	[86.83;107.62]	[−98.91;−25.43]	[112.55;159.87]	[60.60;90.54]	[163.55;222.86]	[−375.13;−202.78]	[220.92;333.58]	[96.89;174.53]	[3.05;3.44]	[0.70;2.08]	[3.03;3.92]	[3.14;3.70]

Note: *p<0.1; **p<0.05; ***p<0.01
