# Supplementary material for: Were the socio-economic determinants of municipalities relevant to the increment of COVID-19 related deaths in Brazil in 2020?
Source: PLoS One. 2022 Apr 28;17(4):e0266109. doi: 10.1371/journal.pone.0266109 (PMC9049518; doi:10.1371/journal.pone.0266109)
Supplement: S2 Table — (PDF) [file pone.0266109.s003.pdf]

Suppl. Table 2. Description of the dimensions of the three socioeconomic status approaches, SVI, HDI, and GeoSES.

Index	Composition of variables
**(a) SVI**	
SVI Urban Infrastructure	% of people living in households with an inadequate water supply and sewage system; % of the population living in urban households without garbage collection service; and % of people living in households with per capita income below half minimum wage (2010) who spend more than one hour to get to work
SVI Human Capital	Mortality up to 1 year of age; % of children aged 0 to 5 years who do not attend school; % of people aged 6 to 14 years who do not attend school; % of women aged 10 to 17 years who had children; % of mothers who are heads of household, without complete fundamental and with a child under 15 years of age; Illiteracy rate of the population aged 15 years or older; % of children who live in households in which none of the residents has complete fundamental education; % of people aged 15 to 24 years who do not study, do not work and have per capita household income equal to or less than half minimum wage (from 2010).
SVI Labor and Income	Proportion of people with per capita household income equal to or less than half the minimum wage (2010); Unemployment rate of the population 18 years old and older; % of people 18 years old and older without complete basic education and in informal jobs; % of people in households with per capita income below half the minimum wage (2010) and dependent on the elderly; and, the activity rate of people 10 to 14 years old.
**(b) HDI**	
HDI Education	Average schooling (in years)
HDI Health	Life expectancy
HDI Income	GDP per capita

(c) GeoSES

GeoSES Education	% of people for whose kind of the highest completed degree was higher education; % of people for whose kind of the highest completed degree was master; % of people whose kind of the highest completed degree was doctorate; % of people whose level of education is unschooled or incomplete Primary school; % of people whose level of education is complete primary school and incomplete high school; % of people whose level of education is complete high school and incomplete higher education; and, % of people whose level of education is complete
GeoSES Poverty	Resident density per room; % of people in poverty line: whose per capita household income per month is less than or equal to R$ 255.00 or US$144.89 (half the minimum wage in 2010); % of people in the poverty line and race, black, brown or indigenous; % of people who in July 2010 had a regular monthly income from the Bolsa Família Social Program or the Child Labor Eradication Program (PETI); % of people who in July 2010 had a regular monthly income from other social programs or transfers

GeoSES Deprivation	% of homes with uncoated masonry; % of households with general sewerage; % of households with general water distribution network; % of households with garbage collected directly by cleaning service; % of households with electricity from electricity distribution company; % of households with TV; % of households with washing machine; % of households with refrigerator; % of households with washing machine, TV and refrigerator; % of households with cell phones; % of households with computer with internet access; % of households with mobile phone and internet computer; % of households with motorcycle for private use; % of households with private car; % of households with adequate housing; % of households with access to sewerage, water supply, garbage collection, electricity and adequate housing; % of households without motorcycles or cars ownerships for private use; % of households with only motorcycles ownership for private use; % of households with only private car ownership
GeoSES Wealth	% of rented households with a rental value of R$1,000.00 (US$ 568.20) or more; %age of households with 4 or more bathrooms; %age of people aged 65 years and over with a monthly income equal to or above R$ 5,100.00 (US$ 2,897.72 or 10 Brazilian minimum wages).
GeoSES Income	Monthly household income in 2010 (R$)

GeoSES Segregation	(number of people with income above R$ 5,400.00 - number of people with income below R$ 1,000.00)/number of respondents; (Number of persons with completed higher education - Number of persons without education and incomplete elementary school)/Total respondents; (number of whites with income over R$ 5,400.00 - number of blacks with income equal to or less than R$ 1,000.00)/Total number of people who answered both questions; (number of whites with income over R$ 5,400.00 - number of black + brown + indigenous with income equal to or less than R$ 1,000.00)/Total number of people who answered both questions; and,(number of whites with income over R$ 5,400.00 - number of whites with income equal to or less than R$ 1,000.00)/total number of people who answered both questions
**(d) Other Indexes**	
Household Per Capita Income	Average monthly family income per capita in 2010
Gini Index	Gini Index of household income
Illiterate Rate	% of illiteracy in the population fifteen years old or older in 2010
Absence of Water and Sanitation	% of resident people without access to water and sewerage
BFP Recipients	% of population benefited by the Bolsa Família Program (BFP)
Child Mortality Rate	Children under 1 year old per 1.000 live births
