# Supplementary material for: Were the socio-economic determinants of municipalities relevant to the increment of COVID-19 related deaths in Brazil in 2020?
Source: PLoS One. 2022 Apr 28;17(4):e0266109. doi: 10.1371/journal.pone.0266109 (PMC9049518; doi:10.1371/journal.pone.0266109)
Supplement: S3 Table — (PDF) [file pone.0266109.s004.pdf]

Suppl. Table 3. Patients description.

Characteristic	N = 607,864[1]
**Gender**	
Men	338,519 (56%)
Women	269,237 (44%)
Unknown	108 (<0.1%)
**Race**	
White	231,095 (38%)
Brown	202,152 (33%)
Unknown	137,849 (23%)
Black	28,209 (4.6%)
Yellow	6,701 (1.1%)
Red	1,858 (0.3%)
**Age**	61 (47, 73)
**Schooling Years**	
Unknown	389,588 (64%)
12 years	69,073 (11%)
5 years	58,244 (9.6%)
9 years	40,265 (6.6%)
Graduated	34,858 (5.7%)
Illiterate	15,836 (2.6%)
**Region**	
Southeast	296,350 (49%)
Northeast	117,219 (19%)
South	83,488 (14%)
Midwest	63,894 (11%)
North	46,913 (7.7%)
**State Capital**	281,773 (46%)
**Area**	
Urban	512,811 (84%)
Unknown	70,236 (12%)
Rural	22,940 (3.8%)
Peri-urban	1,877 (0.3%)

[1]n (%); Median (IQR)
