# Supplementary material for: Were the socio-economic determinants of municipalities relevant to the increment of COVID-19 related deaths in Brazil in 2020?
Source: PLoS One. 2022 Apr 28;17(4):e0266109. doi: 10.1371/journal.pone.0266109 (PMC9049518; doi:10.1371/journal.pone.0266109)
Supplement: Attachment — Submitted filename: PONE-D-21-24701-Editor Comments.pdf [file pone.0266109.s005.pdf]

PLOS ONE

Were the socioeconomic determinants of municipalities relevant to the increment of COVID-19 related deaths in Brazil in 2020?

--Manuscript Draft--

**Manuscript Number:**	PONE-D-21-24701
**Article Type:**	Research Article
**Full Title:**	Were the socioeconomic determinants of municipalities relevant to the increment of COVID-19 related deaths in Brazil in 2020?
**Short Title:**	Socioeconomic determinants did little to explain the municipal COVID-19 mortality rates in Brazil
**Corresponding Author:**	Julio Castro-Alves, Ph.D.<br>Oswaldo Cruz Foundation<br>Rio de Janeiro, RJ BRAZIL
**Keywords:**	COVID-19; SARS-CoV-2; Socioeconomic Status; Social Medicine; Public Health; Epidemiology; Statistical Models; Developing Country; Brazil
**Abstract:**	Background: The COVID-19 pandemic in Brazil has been showing a pattern of distribution of related deaths associated with individual socioeconomic status (SES). However, little is known about the role of SES in the distribution of the mortality rate in different population, from an ecological perspective.<br><br>Objective: The objective of this study was to evaluate the role of socioeconomic factors in the distribution of the COVID-19-related mortality rate among Brazilian municipalities in 2020.<br><br>Methods: We conducted a retrospective, cross-sectional, observational, population-wide, and ecological study, using data of COVID-19-related deaths from the Influenza Epidemiological Surveillance Information System database (SIVEP-Gripe) and SES from the Social Vulnerability Index (SVI), the Human Development Index (HDI), the Geographic Index of the Socioeconomic Context and Social Studies (GeoSES), and 2010 Demographic Census (IBGE/Brazil). We computed crude, age- and sex-standardized, and the latter offset by the time of exposure to the epidemic mortality rates. To determine socioeconomic factors associated with mortality rates we used log-linear models with state codes as a random effect and Haversine variance-covariance matrix.<br><br>Results: 191,528 deaths were related to COVID-19 and distributed in 4,928 (88.55%) Brazilian municipalities. Whatever the socioeconomic indexes used, the R2 were very small to explain SMRT. Consistent across all socioeconomic indexes used, high-income, more educated, and well infrastructure municipalities generally had higher mortality rates.<br><br>Conclusion: Excluding the effect of demographic structure and pandemic timing from mortality rates, the contribution of SES to explain differences in COVID-19-related mortality rates among municipalities in Brazil became very low. The impact of SES on COVID-19-related mortality may vary across levels of aggregation. Urban infrastructure, which includes mobility structures, more complex economic activities and connections, may have influenced the average municipal death rate.
**Order of Authors:**	Julio Castro-Alves, Ph.D.
	Lídia Santos Silva
	João Paulo Lima
	Marcelo Ribeiro-Alves
**Opposed Reviewers:**	
**Additional Information:**	

Powered by Editorial Manager® and ProduXion Manager® from Aries Systems Corporation

Question	Response
**Financial Disclosure**<br><br>Enter a financial disclosure statement that describes the sources of funding for the work included in this submission. Review the submission guidelines for detailed requirements. View published research articles from *PLOS ONE* for specific examples.<br><br>This statement is required for submission and **will appear in the published article** if the submission is accepted. Please make sure it is accurate.<br><br>**Unfunded studies**<br>Enter: *The author(s) received no specific funding for this work.*<br><br>**Funded studies**<br>Enter a statement with the following details:<br>• Initials of the authors who received each award<br>• Grant numbers awarded to each author<br>• The full name of each funder<br>• URL of each funder website<br>• Did the sponsors or funders play any role in the study design, data collection and analysis, decision to publish, or preparation of the manuscript?<br>• **NO** - Include this sentence at the end of your statement: *The funders had no role in study design, data collection and analysis, decision to publish, or preparation of the manuscript.*<br>• **YES** - Specify the role(s) played.<br><br>* typeset	This research was funded by Inova-Fiocruz Program (VPPCB-005-FIO-20-2-45, https://portal.fiocruz.br/programa-inova-fiocruz) to M. Ribeiro-Alves and Inova-Fiocruz Program (VPPCB-005-FIO-20-2-61, https://portal.fiocruz.br/programa-inova-fiocruz) to J. Castro-Alves. The funders had no role in the conceptualization, analysis, interpretation, or decision to publish this manuscript.
**Competing Interests**<br><br>Use the instructions below to enter a competing interest statement for this submission. On behalf of all authors, disclose any competing interests that could be perceived to bias this work—acknowledging all financial support and any other relevant financial or non-financial competing interests.	The authors have declared that no competing interests exist.

Powered by Editorial Manager® and ProduXion Manager® from Aries Systems Corporation

This statement is required for submission and **will appear in the published article** if the submission is accepted. Please make sure it is accurate and that any funding sources listed in your Funding Information later in the submission form are also declared in your Financial Disclosure statement.<br><br>View published research articles from *PLOS ONE* for specific examples.<br><br>**NO authors have competing interests**<br><br>Enter: *The authors have declared that no competing interests exist.*<br><br>**Authors with competing interests**<br><br>Enter competing interest details beginning with this statement:<br><br>*I have read the journal's policy and the authors of this manuscript have the following competing interests: [insert competing interests here]*<br><br>* typeset	
**Ethics Statement**<br><br>Enter an ethics statement for this submission. This statement is required if the study involved:<br><br>• Human participants<br>• Human specimens or tissue<br>• Vertebrate animals or cephalopods<br>• Vertebrate embryos or tissues<br>• Field research<br><br>Write "N/A" if the submission does not require an ethics statement.<br><br>General guidance is provided below. Consult the submission guidelines for detailed instructions. **Make sure that all information entered here is included in the Methods section of the manuscript.**	N/A

Powered by Editorial Manager® and ProduXion Manager® from Aries Systems Corporation

**Format for specific study types**<br><br>**Human Subject Research (involving human participants and/or tissue)**<br>• Give the name of the institutional review board or ethics committee that approved the study<br>• Include the approval number and/or a statement indicating approval of this research<br>• Indicate the form of consent obtained (written/oral) or the reason that consent was not obtained (e.g. the data were analyzed anonymously)<br><br>**Animal Research (involving vertebrate animals, embryos or tissues)**<br>• Provide the name of the Institutional Animal Care and Use Committee (IACUC) or other relevant ethics board that reviewed the study protocol, and indicate whether they approved this research or granted a formal waiver of ethical approval<br>• Include an approval number if one was obtained<br>• If the study involved *non-human primates*, add *additional details* about animal welfare and steps taken to ameliorate suffering<br>• If anesthesia, euthanasia, or any kind of animal sacrifice is part of the study, include briefly which substances and/or methods were applied<br><br>**Field Research**<br><br>Include the following details if this study involves the collection of plant, animal, or other materials from a natural setting:<br>• Field permit number<br>• Name of the institution or relevant body that granted permission	
**Data Availability**<br><br>Authors are required to make all data underlying the findings described fully available, without restriction, and from the time of publication. PLOS allows rare exceptions to address legal and ethical concerns. See the PLOS Data Policy and FAQ for detailed information.	Yes - all data are fully available without restriction

Powered by Editorial Manager® and ProduXion Manager® from Aries Systems Corporation

A Data Availability Statement describing where the data can be found is required at submission. Your answers to this question constitute the Data Availability Statement and **will be published in the article**, if accepted.<br><br>**Important:** Stating 'data available on request from the author' is not sufficient. If your data are only available upon request, select 'No' for the first question and explain your exceptional situation in the text box.<br><br>Do the authors confirm that all data underlying the findings described in their manuscript are fully available without restriction?	
**Describe where the data may be found in full sentences. If you are copying our sample text, replace any instances of XXX with the appropriate details.**<br><br>• If the data are **held or will be held in a public repository**, include URLs, accession numbers or DOIs. If this information will only be available after acceptance, indicate this by ticking the box below. For example: *All XXX files are available from the XXX database (accession number(s) XXX, XXX.*).<br>• If the data are all contained **within the manuscript and/or Supporting Information files**, enter the following: *All relevant data are within the manuscript and its Supporting Information files.*<br>• If neither of these applies but you are able to provide **details of access elsewhere**, with or without limitations, please do so. For example:<br><br>*Data cannot be shared publicly because of [XXX]. Data are available from the XXX Institutional Data Access / Ethics Committee (contact via XXX) for researchers who meet the criteria for access to confidential data.*<br><br>*The data underlying the results presented in the study are available*	The data underlying the results presented in the study are available from Influenza Epidemiological Surveillance Information System database, InfoGripe, Brazilian Ministry of Health (https://opendatasus.saude.gov.br/dataset/ae90fa8f-3e94-467e-a33f-94adbb66edf8).

Powered by Editorial Manager® and ProduXion Manager® from Aries Systems Corporation

from (include the name of the third party and contact information or URL).<br>• This text is appropriate if the data are owned by a third party and authors do not have permission to share the data.<br><br>* typeset	
Additional data availability information:	

Powered by Editorial Manager® and ProduXion Manager® from Aries Systems Corporation

Ministério da Saúde

FIOCRUZ
Fundação Oswaldo Cruz

27th July, 2021

To: Editor-in-Chief

PLoS ONE Journal

We would be grateful if you consider the manuscript entitled "**Were the socioeconomic determinants of municipalities relevant to the increment of COVID-19 related deaths in Brazil in 2020?"** for publication in **PLoS ONE Journal.**

In our retrospective, cross-sectional, observational, population-wide, and ecological study, we evaluated the importance of municipal socioeconomic factors as a determinant of COVID-19-related mortality rate in Brazil in 2020. ROUBAUD et al. (2020) and CASTRO et al. (2021) also used a similar study design and data, however, we utilized a wider time span, three different definitions of mortality rate (crude, sex- and age-standardized, and weighted by the time of the pandemic), as well as four socioeconomic status indexes in the analyses. Our research concluded that, consistent across all socioeconomic indexes tested, high-income, more educated, and well infrastructure municipalities generally had higher mortality rates. Differently from what was hypothesized, socioeconomic factors explained very little the differences in municipal mortality rates observed in the pandemic COVID-19 in Brazil. We know, however, that these results cannot be directly reflected in the reality of individual and/or intra-municipal clusters.

This study was funded by Inova Oswaldo Cruz Foundation Program, grant numbers: VPPCB-005-FIO-20-2-45 and VPPCB-005-FIO-20-2-61. These funders had no role in study design, data collection and analysis, decision to publish, or preparation of the manuscript.

The data, models, or methodology used in the research are not proprietary. This manuscript has not been published and is not under consideration for publication elsewhere. We have no conflicts of interest to disclose related to this topic. All authors contributed significantly to the study and approved the final version of the manuscript as well as this submission.

Av. Brasil, 4365 - Manguinhos, Rio de Janeiro CEP: 21040-360 Tel.:(0xx21) 3865-9595
e-mails: marcelo.ribeiro@fiocruz.br / marcelo.ribeiro@ini.fiocruz.br /
mribalves@gmail.com

Sincerely,

Dr. Julio Castro-Alves

Fundação Oswaldo Cruz

Av. Brasil, 4365 - Manguinhos, Rio de Janeiro CEP: 21040-360 Tel.:(0xx21) 3865-9595
e-mails: marcelo.ribeiro@fiocruz.br / marcelo.ribeiro@ini.fiocruz.br /
mribalves@gmail.com

Manuscript Click here to access/download;Manuscript;manuscript.pdf

Were the socio-economic determinants of municipalities relevant to the increment of COVID-19 related deaths in Brazil in 2020?

Julio Castro-Alves[1*], Lídia Santos Silva[1], João Paulo Lima[1], Marcelo Ribeiro-Alves[1],

1 Instituto Nacional de Infectologia Evandro Chagas, Fiocruz. Rio de Janeiro, RJ. Brazil

* corresponding author Julio.castro.alves.lima@gmail.com

Abstract

Background: The COVID-19 pandemic in Brazil has been showing a pattern of distribution of related deaths associated with individual socioeconomic status (SES). However, little is known about the role of SES in the distribution of the mortality rate in different population, from an ecological perspective. Objective: The objective of this study was to evaluate the role of socioeconomic factors in the distribution of the COVID-19-related mortality rate among Brazilian municipalities in 2020. Methods: We conducted a retrospective, cross-sectional, observational, population-wide, and ecological study, using data of COVID-19-related deaths from the Influenza Epidemiological Surveillance Information System database (SIVEP-Gripe) and SES from the Social Vulnerability Index (SVI), the Human Development Index (HDI), the Geographic Index of the Socioeconomic Context and Social Studies (GeoSES), and 2010 Demographic Census (IBGE/Brazil). We computed crude, age- and sex-standardized, and the latter offset by the time of exposure to the epidemic mortality rates. To determine socioeconomic factors associated with mortality rates we used log-linear models with state codes as a random effect and Haversine variance-covariance matrix. Results: 191,528 deaths were related to COVID-19 and distributed in 4,928 (88.55%) Brazilian municipalities. Whatever the socioeconomic indexes used, the R^2 were very small to

explain SMRT. Consistent across all socioeconomic indexes used, high-income, more educated, and well infrastructure municipalities generally had higher mortality rates. Conclusion: Excluding the effect of demographic structure and pandemic timing from mortality rates, the contribution of SES to explain differences in COVID-19-related mortality rates among municipalities in Brazil became very low. The impact of SES on COVID-19-related mortality may vary across levels of aggregation. Urban infrastructure, which includes mobility structures, more complex economic activities and connections, may have influenced the average municipal death rate.

Introduction

In December 2019, in Wuhan (China), the first cases of infection with the SARS-CoV-2 virus emerged [1]. By January 30th, 2020, the date it was declared by the World Health Organization (WHO) as a Public Health Emergency of International Importance [2], the new coronavirus had already spread to 18 countries. The contamination and deaths from COVID-19 accelerated, forcing the WHO, on March 11th, 2020, to declare a situation of Pandemic [2].

Despite poverty and inequality have not been prominent causal factors in epidemiological transmission models for COVID-19, the social science literature has pointed to many ways in which the two can be expected to result in increased vulnerability to coronavirus infection [3]. From the perspective of social epidemiology [4], a system of inequality containing social aspects such as education, material conditions and infrastructure, poor working conditions, racial segregation, and gender inequality, acts simultaneously in cascade influencing the biology of disease and death. A general premise of this line of thinking is that social inequalities generate health inequalities, or that socioeconomic inequalities would shape individuals' exposure and susceptibility to SARS-CoV-2 infection and COVID-19-related deaths. Based on this thinking, Clouston et al. (2021) proposed that the spread pattern of the COVID-19 epidemic would have three stages. In the first stage, the early period of epidemic when SARS-CoV-2 virus emerge and diffuse through the population [5]. At first, elites and high socioeconomic status (SES) communities would become infected because they were not yet prepared to deal with the emerging disease, leading initial cases to occur in

higher SES locations, and, gradually, spreding to the community, which includes lower SES. It is inline with the evidence in Rio de Janeiro, Brazil, as we observed this behavior in the early months of the pandemic, when age standardized incidence rates were higher in wealthy neighbourhoods [6]. In a second stage, as a public health response arises, communities begin to implement social distancing strategies to control the risk of viral spread and contamination, inequalities emerge and grow. Due to the distinct ability to mobilize resources in containing the spread of the virus, high and low SES communities would face divergent epidemic situations. For the former, there would be a reduction in prevalence and a gradual decrease in the risk of COVID-19-related death. For the latter, the burden of disease would increase and they would experience more and more cases of COVID-19. The health inequalities would arise as an unequal diffusion of innovations. Collectively, poorer people tend to be the ones most harmed by measures restricting social interaction, consumption, and the circulation of goods imposed by countries, states, and municipalities, even more so if ~~lockd-owns~~ are imposed; like the ones widely instituted by European countries. Furthermore, even though poorer people are more likely to continue working outside the home during a pandemic [7], the economic crisis caused by the restriction of consumption by the higher-income population would prevent much of the low-income population from having access to a job, which results in involuntary isolation not only by the absence of work, but also by the consequent decrease in consumption [8]. Regarding morbidity and mortality, many of the risk factors associated with COVID-19 severity and death may also be related to income. Poorer people tend to have higher rates of malnutrition, tend to develop more and earlier chronic diseases such as obesity, type II diabetes mellitus, systemic arterial hypertension, and tend to have less access to health care, generally of lower quality. As the pandemic progresses, the presence of a health gradient against racial minorities, black/brown people [9–12], vulnerable populations, low per capita income, and population living in an overcrowded household [11–14] become even more evident. Even in wealthy countries such as the United States and England there were higher rates of COVID-19-related deaths in less favored areas [15, 16]. In Brazil, Baqui et al. (2020) showed that black/brown individuals were more affected due to the difficulty of access to health care [17], while others have confirmed that workers in essential functions, dependent on mass public transportation, were highly exposed to

the virus [13, 18]. Finally, the third stage would be characterized by reduction of health inequalities due to the increasing access to health knowledge among populations, just as with the development of effective vaccines, and the eventual disease elimination. The availability of vaccines against the SARS-CoV-2 virus became a reality in 2021, although total eradication or elimination in countries depends on the speed of vaccination and the immunization coverage achieved in the population.

The Clouston et al. (2021) narrative about the rise and fall of health inequalities during the COVID-19 epidemic and their association with SES inequalities is quite consistent considering the Brazilian experience regarding intra-municipal (or individual) data [5, 6, 17–21]. However, in relation to the municipalities, the inter-municipal or ecological dynamics of spread, another narrative seems to be prevailed. Being a continental and very unequal country, we had in Brazil not one, but several patterns of COVID-19 spread. It was observed that each federative unit, or macroregion, differed from each other in relation to the beginning of the pandemic. The first case of contamination of the new coronavirus in Brazil was recorded on February 26th in São Paulo [22], and in the neighboring state, Rio de Janeiro, the first record occurred in early March [20]. From the wealthier southeastern region, the pandemic migrated to the poorer northern region, reaching it on March 13th, in Manaus, in the state of Amazonas [23], in the state of Acre in mid-March [24], and in the state of Roraima in April [25]. The SARS-CoV-2 virus circulated first among densely populated municipalities (capital cities) and, gradually, due to their dense urban network, that also connects to other municipalities, spread to other capital cities and, from these, to the interior of the country [26–28]. Consequently, urban infrastructure, which includes mobility structures, roads, transportation, more complex economic activities and connections, and income, have influenced the average municipal death rate. In fact, in ecological studies even in other countries in the world, such as France, a similar inter-municipal pattern has been observed [13].

The role of socioeconomic status as a determinant of mortality inequality during the COVID-19 pandemic in Brazil seems to diverge depending on the level of the analysis, which raises the need to enlarge the understanding of this relationship from an ecological perspective. So far, no study used a convenient definition of the mortality rate and/or used data from the entire Brazilian territory to compare different regions of

Brazil. Studies using data from Brazilian regions have shown that the increase in SES, represented by the human development index, was associated with an average decline in crude mortality rate [29–31]. Other studies analyzed the municipal level data in Brazilian territories but using a small sample of municipalities, which provide us a biased source of information [32]; [33]; [28]. The standardized mortality rate includes in its calculation the demographic structure of the region, normalizing it. It is also essential to control the timing of the epidemic in the mortality rate. This can be done in studies involving one or more municipalities of the same region that had the first records of cases in very early epidemiological weeks [20], or even, as we chose, controlling the mortality rate by the time of the pandemic in each region. Altogether, and since the COVID-19 spread was from the capitals to the interior, we chose to use the standardized mortality rate adjusted by the pandemic time of each municipality, nationwide. In addition, there is a lack of studies investigating the role of socioeconomic inequalities using a robust set of SES indices to explain differences in mortality rates among regions.

The aim of this study was to examine the role of socioeconomic factors in the distribution of the COVID-19-related mortality rate among Brazilian municipalities in 2020. To this end, we calculate age- and sex-standardized, time-adjusted mortality rates of COVID-19 for all municipalities from February 20th to December 31st, 2020. Furthermore, we develop a robust analysis comparing different definitions of mortality rate and a broad set of available SES indices.

Material and Methods

Data

We conducted a retrospective, cross-sectional, observational, population-wide, and ecological study of the relationship between socioeconomic status and the COVID-19-related mortality rate by municipality of residence. Below, we briefly describe the data and information sources ~~employed~~.

Health Data

The information on the number of COVID-19-related deaths and the pandemic time (date of first symptoms for the first recorded case) by the municipality of residence was extracted from the Influenza Epidemiological Surveillance Information System database (SIVEP-Gripe), as of January 11, 2021 (Downloaded from https://opendatasus.saude.gov.br/dataset/ae90fa8f-3e94-467e-a33f-94adbb66edf8). SIVEP-Gripe is an official open-source national surveillance database used to monitor severe acute respiratory infections in Brazil and resulting hospital admissions, established and updated by the Brazilian Ministry of Health (MoH, Brazil). From this, we selected only patients with a confirmed diagnosis of COVID-19 (laboratory, clinical, clinical-epidemiological, or imaging criteria) with clinical evolution until COVID-19-related death from February 20 (first confirmed case of COVID-19 in Brazilian territory) to December 31, 2020. Next, the number of deaths and the pandemic time until December 31, 2020, were grouped by the municipality of residence of each registry.

Socioeconomic Data

For us to assess the robustness of our observed results, we used four different ways to quantify the socioeconomic status. These were: the Social Vulnerability Index (SVI) (http://ivs.ipea.gov.br/index.php/pt/), developed by the Institute for Applied Economic Research (IPEA, Brazil) (https://www.ipea.gov.br/portal/index.php?option=com_content&id=19153); the Human Development Index (HDI), developed by the United Nations (UN); the Geographic Index of the Socioeconomic Context and Social Studies (GeoSES) [34] (https://doi.org/10.1371/journal.pone.0232074.s003), developed by the Support Program for Institutional Development of the Unified Health System (PROADI-SUS) and coordinated by the Unified Health System (SUS) Monitoring and Evaluation Department (DEMAS/MoH, Brazil); and, finally, a selection of socioeconomic indices/rates among many developed by the Brazilian Institute of Geography and Statistics (IBGE, Brazil). The four socioeconomic approaches were created from variables from the 2010 Demographic Census covering all Brazilian municipalities

(IBGE, Brazil). Below, we will further detail each of these theoretical ways of conceiving the socioeconomic status of Brazilian municipalities. Detailed information about each index composition can be found at supplemental table 2.

The SVI is based on the recognition that social vulnerabilities arise from broader social processes, which individuals have little autonomy to change, and therefore, it would be up to the State to change these conditions through public policies. In this sense, the index is separated into three large sets of assets, whose possession, or deprivation, would determine the welfare conditions of the populations: Urban Infrastructure, Human Capital, and Income and Labor. Each of these dimensions varies from 0 to 1, where 0 corresponds to the ideal situation and 1 corresponds to the worst situation, that is, municipalities with the SVI close to 1 are in the situation of maximum social vulnerability. The aggregated SVI index would be a composition of these three dimensions, varying in the same range.

The Human Development Index (HDI) is intended to measure the degree of human development of a population, defined as the range of choices offered in the course of life and the freedom for individuals to enjoy the life they desire. This freedom and range of choices are greatly influenced, of course, by the length of their lives, access to ~~knowledge~~, and material standard of living. The HDI is therefore a summary measure of three basic dimensions: health, education, and income. The aggregate HDI, as well as its three dimensions, is represented by a number ranging from 0 to 1; the closer to 1, the greater the human development of the population.

The Socioeconomic Index of the Geographic Context for Health Studies (GeoSES) aims to evaluate and monitor health inequalities in Brazil. For this, it is based on the assumption that the socioeconomic status (SES) is related to the prevalence of several diseases, the disease gradient, and that the socio-economic context where individuals are inserted is essential to understand health inequalities. In this sense, the index summarizes the living conditions in that locality, separated into seven dimensions: education, poverty, wealth, income, racial segregation and deprivation, and services. The GeoSES Income index increases as the average wage of the employed population increases. The GeoSES Education index decreases as the educational attainment of the population increases. GeoSES Poverty decreases when the poverty of the population increases. GeoSES Wealth seeks to represent the stock of resources in a given locality

through real estate information, and the index grows as wealth increases in the municipality. The GeoSES Material Deprivation is measured through the material resources and conveniences available (such as adequate housing, ownership of a car, refrigerator, computer, etc.), also assessing their access to services such as sanitation, electricity, and internet. The higher the GeoSES Material Deprivation, the greater the material deprivation that a given municipality's population goes through. Finally, GeoSES Segregation considers aspects of inequality in access to education and income, including income and education levels stratified by ethnic groups. The higher the segregation in a municipality, the lower the GeoSES Segregation.

The fourth socioeconomic environment evaluated was made up of the variables Per Capita Household Income (average monthly family income per capita in 2010), Gini Index of household income, Illiteracy Rate (% of illiteracy in the population fifteen years old or older in 2010), Absence of Water and Sanitation (% of resident people without access to water and sewerage), Bolsa Família Program (BFP) Beneficiary (% of population benefited by the BFP), and Infant Mortality Rate (children under 1 year old per 1.000 live births), obtained from the 2010 census (IBGE/Brazil), and not directly considered in the composition of the composite indexes SVI, HDI, or GeoSES.

Statistical Modeling

We computed three distinct mortality rates (per 100,000 people) by municipalities in 2020: crude mortality rate (CMR), age and sex standardized mortality rate (SMR), and age and sex standardized mortality rate offset by the time of exposure to the epidemic (SMRT). CMR was calculated as the number of deaths from COVID-19 by the total population exposed to the risk (total municipality population) in 2010. SMR had computation similar to CMR, but each age-specific mortality rate by municipality was standardized (re-weighted) by using the Brazilian population pyramid (age and gender pyramid) in 2010 as reference. Lastly, SMRT is equal to SMR divided by the time of exposure to the epidemic, the last defined as the time (in months) since the first positive case of SARS-CoV-2 infection in each specific municipality.

To determine socioeconomic factors associated with mortality rates we used log-linear models adopting state codes as a random effect for the intercept. The three

definitions of mortality rate were used as outcomes and the four groups of socioeconomic indexes as independent variables. The models were adjusted for Crowding Rate (%age of the population living in a house with more than two people per room in 2010), Demographic Density Rate (number of inhabitants per km2 in 2010), State Capitals, and Travel Time to State Capital (in hours). We also did robustness checks using health infrastructure, proxied by ICU Hospital Beds Rate (the number of hospital beds in ICU per million inhabitants in 2015) and Physician Rate (number of physicians per thousand inhabitants in 2015) as controls. Measures of association were presented in terms of % change (exponentiated value of geometric mean) adjusted for confounders. The independence assumption among municipalities was loosened by including a Haversine variance-covariance matrix, where the dependence among municipalities (covariance) is computed by the spatial correlation. The Haversine formula calculated the geodesic distance between two municipalities using points specified by radian latitude/longitude. We used the 35) method to obtain the explained variance coefficient (R^2) from generalized linear mixed-effects models, partitioned in two definitions. The marginal R^2 represents the proportion of variability explained by the fixed effects, and the conditional R^2 is the proportion of total variance explained through both fixed and random effects. All statistical analyses were performed in R v.4.0.5 software, utilizing the 'nlme' and 'sf' libraries and their dependencies to develop statistical models and maps, respectively.

Results

Sample Description

Of the 1,136,681 records present in the severe acute respiratory syndrome database (SIVEP-Gripe/MoH) as of January 11th, 2021, 109 were eliminated for not having geographic information (country, state, city) of record origin, 2,681 for having a negative age, and 202 for being older than 105 years. In addition, in the remaining 1,133,689 records, the year, less than 2019 or greater than 2021, was corrected for dates of case notification, first symptoms, start and end of hospitalization, admission and discharge from intensive care units, clinical course, and death. Of the remaining 1,133,689 records,

606,554 (53.5%) had SARS-CoV-2 infection confirmed by laboratory, clinical, clinical-epidemiological, or clinical imaging (lung X-ray/computed tomography) criteria, and 191,528 (16.89%) had a death likely related to COVID-19 by that date. These deaths were distributed in 4,928 (88.55%) of the 5,565 Brazilian municipalities (IBGE); only 14 of these municipalities had recorded cases of COVID-19 without any deaths.

Figure **??** presents the distribution of the mortality rate among Brazilian municipalities according to the three different definitions, (A) CMR, (B) SMR, and (C) SMRT, as well as (D) a tabulation containing median values of these rates divided by Regions and sub-divided between state capital and non-capital municipalities. This figure allows the observation of two different aspects of mortality in Brazil over the course of the 2020 COVID-19 pandemic, which must be considered together. The first is that, regardless of the definition of mortality rate, the figures are appropriate for assessing relative mortality among different regions and/or municipalities in the Brazilian territory. Second, is that the different mortality rate definitions allow us to evaluate changes in the increments of these rates given either by regional differences, or even, between state capitals and non-capital municipalities, of the age and gender distributions of the populations, or, no less discrepant, of the epidemic timing experienced by these municipalities/regions in the year 2020. Regardless of the death rate definition, the North region was the most affected, followed closely by the Midwest region, while the Northeast region was the least affected. The state capital municipalities were always more affected than non-capitals, either nationally or regionally. Apparently, the use of different death rate definitions seems to influence the relative severity among municipalities. For example, when comparing between the CMR (Figure 1-A) and the SMR (Figure 1-B) we notice a worsening in the latter in the North and Central-West regions, possibly due to the greater contribution of young people in the demographic composition of these regions. When we compare the SMR (Figure 1-B) and the SMRT (Figure 1-C), we see a decrease in severity, still high, in the coastal region, where most Brazilian state capitals are concentrated, possibly due to the longer duration of the epidemic in this region. The tabulation (Figure 1-D) confirms these movements. There was an average 2.48-fold increase between CMR and SMR median mortality rates in the North and Midwest regions, while for the Northeast, Southeast and South regions this average increase was only 1.83-fold. Similarly, we observed an

average increase of 2.82 times between the median mortality of capital and non-capital municipalities by the SMR mortality rate, while we observed an average increase of 1.86 times between the same municipalities using the median SMRT mortality rates.

INSERT FIGURE 1 HERE

Figure 1 - Brazilian National Map by Municipalities with Distribution of Mortality Rates (a, b and c) and Medians of Mortality Rate by Geographic Locations (d). Brazil, 2020.

Table 1 shows the median SMRT and interquartile ranges of the municipalities included in the top 10 and bottom 10 percentiles (10th or 1st decile) of different socioeconomic dimensions of Brazilian municipalities. The table focuses attention on the municipalities at the extremes of the distributions of socioeconomic dimensions, that is, how the top 10% municipalities differ from the bottom 10% considering each socioeconomic attribute in terms of SMRT. The inequality in the distribution of SMRT among Brazilian municipalities is evident when we look at the extreme deciles. The municipalities in the first SVI decile, or those with the highest social vulnerability, had a median SMRT of 18.46 (IQR = 17.26) while, at the other extreme, or the municipalities with the lowest social vulnerability, the median was 13.52 (IQR = 18.73). With respect to HDI, while the 10% worst municipalities in terms of human development had a median SMRT of 12.86 (IQR = 16.2), the 10% municipalities with the best human development indexes had a median SMRT of 19.95 (IQR = 16.47). Finally, the 10% municipalities with the lowest and highest socioeconomic status in the GeoSES had SMRT of 14.02 (IQR = 17.59) and 19.54 (IQR = 16.66), respectively. We observed in this preliminary examination, according to the three aggregate socioeconomic status indexes, that municipalities with higher socioeconomic levels had on average a higher SMRT than the relatively less developed municipalities, despite the large variability indicated by the wide IQR ranges. A similar situation was monotonically observed for the socioeconomic status. For example, the first decile of municipalities with the lowest inequality rates as measured by the Gini Index had a median of 19.81 (IQR = 20.05), while the tenth decile of municipalities with the highest inequality rates had a median of 16.97 (IQR = 23.53). It is worth noting that, as observed in the tenth deciles of the Gini, GeoSES Income and Household Per Capita Income Indexes, the largest interquartile ranges of the GeoSES Poverty, Deprivation and

Segregation Indexes were also larger in the deciles representing better social conditions, first deciles for the latter, suggesting a greater variability in the SMRT distribution among the most developed Brazilian municipalities.

Table 1. Descriptive Analysis of SMRT by Botton and Top Deciles of Attributes of Brazilian Municipalities. Brazil, 2020.

	Municipality Deciles by SMRT	
	1st (0-10%) median (IQR)	10th (90-100%) median (IQR)
**Socioeconomic Variables**		
SVI	18.46 (17.26)	13.52 (18.73)
SVI Urban Infrastructure	18.72 (21.15)	16.22 (20.23)
SVI Human Capital	19.18 (16.12)	14.69 (18.18)
SVI Labor and Income	19.69 (16.53)	11.75 (14.38)
HDI	12.86 (16.2)	19.95 (16.47)
HDI Education	14.15 (16.44)	20.1 (16.57)
HDI Health	13.66 (14.77)	18.24 (17.07)
HDI Income	12.44 (15.3)	19.51 (16.69)
GeoSES	14.02 (17.59)	19.54 (16.66)
GeoSES Education	20.45 (16.03)	13.67 (14.81)
GeoSES Poverty	19.89 (16.52)	13.52 (16.46)
GeoSES Deprivation	14.25 (18.42)	19.72 (17.72)
GeoSES Wealth	16.88 (20.45)	18.24 (16.51)
GeoSES Income	11.64 (12.78)	20.32 (17.7)
GeoSES Segregation	14.48 (15.85)	18.58 (18.58)
Household Per Capita Income	13.09 (15.12)	19.97 (17.27)
Gini Index	19.81 (20.05)	16.97 (23.53)
Illiterate Rate	20.79 (16.99)	12.08 (13.51)
Absence of Water and Sanitation	17.64 (18.67)	14.12 (19.11)
BF Recipients	20.12 (16.75)	12.74 (13.77)
Child Mortality Rate	19.44 (20.48)	16.48 (21.05)
**Control Variables**		
Crowding Rate	19.1 (19.95)	18.63 (23.23)
Demographic Density Rate	20.48 (25.54)	24.42 (16.28)
Travel Time	27.28 (19.61)	13.73 (16.47)
Physician Rate	18.84 (21.2)	16.42 (16.21)
ICU Hospital Beds Rate	15.82 (17.58)	20.82 (16.47)

Relationship between different socioeconomic dimensions and COVID-19-related mortality rate

Table 2 presents the estimated parameters in mixed-effects log-linear models and their marginal and conditional coefficients of determination (R2) for the definitions of mortality rate (CMR, SMR, and SMRT), dependent variables, with the dimensions of

the composite socioeconomic indexes (SVI, HDI, and GeoSES) and other socioeconomic indicators, independent variables; totaling twelve distinct models (Table 2 (1)-(12)), of same random effect and same spatial dependence structure.

In order to deepen the relationship between the various socioeconomic dimensions/indicators of the Brazilian municipalities with the mortality rate, we chose both to use the disaggregated version of the composite indexes, and to focus our presentation of results on the SMRT mortality rate, as we consider it more "fair"; since it corrects and standardizes the age distribution of the population by sex, as well as the pandemic time expected by each municipality. The results with the CMR and SMR definitions are used in comparison to the SMRT when relevant.

Regarding the socioeconomic dimension of education (Table 2), for the HDI Education, we observed a direct (positive) and significant (P-value < 0.05) relationship with all the mortality rate definitions, i.e., the more educated the municipality's population was, the higher its COVID-19 mortality rate was; albeit independently of the age distribution by sex or the pandemic time expected by that municipality. Consistently, the lower a municipality's Illiteracy Rate was the higher the observed SMRT was (P-value < 0.01). For two other composite index dimensions intrinsically associated with education, SVI Human Capital and GeoSES Education, we observed signs of the mean negative and positive adjusted coefficients, respectively, consistent with those previously cited, however, not significant in models with the SMRT outcome. Remember that, while an increase in the value of any SVI dimension indicates greater vulnerability, the higher the observed value of a municipality's GeoSES Education index, the lower the expected level of schooling for its population.

Regarding the socioeconomic dimension of income and poverty (Table 2), in general, we observed that the lower-income municipalities were not those for which we observed the highest death rate in the year 2020, even though controlled by the other socioeconomic dimensions of the same composite indices and by factors intrinsic to the Sars-Cov-2 spread, that is, the distance between the observed municipality and the state capital (common origin of the pandemic in each state) or between nearby municipalities, population density, political and climatic conditions, and other characteristics of each Brazilian state. The increase in SVI Income and Labor was indirect, negative coefficients, significantly (P-value < 0.01) related to the increase in SMRT. Consistently,

increases in Household Per Capita Income and HDI Income were directly related, positive coefficients, significantly (P-value < 0.01) to increases in SMRT. Recall that these three results indicate that the higher the income, i.e., the lower the SVI Income and Labor, and the higher the Household Per Capita Income and HDI Income, the higher the SMRT corrected death rate. The composite GeoSES index includes the dimensions GeoSES Income, directly associated with income, and two other GeoSES Wealth and Poverty more associated with poverty. While for the former we observed no significant relationship with SMRT, increasing GeoSES Wealth was indirect, negative sign, significantly related to increasing SMRT (P-value < 0.01). The same, indirect relationship, was observed for GeoSES Poverty not only with significantly increased SMRT but also with that of CMR and SMR (P-values < 0.01). These two dimensions of GeoSES agree that the higher the wealth or lower the poverty of a municipality, the higher the average COVID-19-related mortality rate experienced by that municipality in Brazil in 2020. Finally, we observed no significant relationship between the BFP Recipient population, published by IBGE, and the SMRT. This last result may somehow indicate the efficiency of social security programs, that is, we could not interpret it as increased in poorer municipalities, but rather interpret it as municipalities where the social safety net is more efficient regardless of higher or lower income.

Still related to income and poverty, but measuring intraregional or inter-municipal inequality of these two, we respectively observed the relationships of both the Gini index, published by IBGE, and the Segregation dimension of the GeoSES composite index with the different definitions of mortality rate (Table 2). The increase in both was related indirectly, negative coefficients, and significantly with the increase in SMRT (P-value < 0.01). That is, not only did richer and less poor municipalities have higher SMRT on average, but those with higher income inequality and/or poverty were the ones that experienced higher COVID-19-related death rates. Recalling that the higher, or closer to 1, is the Gini index the greater the inequality in income distribution, and similarly, the higher the GeoSES Segregation measure the less segregation, understood as the separate housing of different population groups in different parts of a city or segregation areas, observed in that municipality.

Regarding the dimensions directly associated with health and infrastructure (Table 2), the HDI Health index and SVI Infrastructure, we observed conflicting and

significant results only after corrections and standardization by the population age distribution by sex and by the pandemic time expected by each municipality, i.e., for the SMRT outcome and not for the CMR and SMR outcomes. We observed both an indirect relationship, negative coefficients, with the SMRT. That is, the better the health status, or even, the higher the life expectancy experienced in a municipality, the lower on average was the observed SMRT (P-value < 0.01), whereas the higher the vulnerability of a municipality's infrastructure, that is, the lower the conditions of access to basic sanitation and urban mobility services, the lower, on average, was the observed SMRT. We did not observe a significant relationship between SMRT and GeoSES Deprivation, which very close to SVI Infrastructure would indicate the deprivation of material resources and essential services of a municipality, thus we cannot assess the robustness of this observation; we can only state that the higher the GeoSES Deprivation the lower was its crude mortality rate, or CMR (P-value < 0.01), to some degree corroborating the SVI Infrastructure results. Remembering that for any dimension of SVI, the greater the increase in the value of GeoSES Deprivation, the greater the deprivation of the measured region.

This much said, when we analyze the values of the coefficients of determination, R^2, especially those related to the socioeconomic dimensions/indicators of the Brazilian municipalities, that is, the marginal coefficient of determination of each log-linear mixed effect model, we have results that vary from 0.042 (4.2%), obtained with the inclusion of the socioeconomic dimensions of the HDI composite indicator, up to 0.169 (16.9%), obtained with the inclusion of the variables Per Capita Household Income, Gini Index of household income, Illiteracy Rate, Absence of Water and Sanitation, Bolsa Família Program (BFP) Beneficiary, and Child Mortality Rate, obtained from the 2010 census (IBGE/Brazil) (Table 2). That is, while we were able to point to robust municipal patterns regarding their socioeconomic conditions associated COVID-19-related mortality rate in Brazil in 2020, we also observe that average municipal socioeconomic information explains very little, whatever it is, variation in the COVID-19-related mortality rate in Brazil in 2020.

Table 2. Adjusted Log-linear Models to Estimate Association Between COVID-19 Mortality Rate (CMR, SMR and SMRT) and Socioeconomic Status in Brazilian Municipalities. Brazil, 2020.

	Dependent variable:											
	CMR				SMR				SMRT			
	(1)	(2)	(3)	(4)	(5)	(6)	(7)	(8)	(9)	(10)	(11)	(12)
SVI Human Capital	−23.535**				−17.831				−0.065			
SVI Labor and Income	−62.866***				−260.769***				−1.103***			
SVI Urban Infrastructure	−7.902				−5.536				−0.165*			
HDI Education		28.291**				79.863***				0.606**		
HDI Health		−12.968				−55.835				−0.892*		
HDI Income		176.743***				530.989***				1.946***		
GeoSES Education			−0.391***				−1.099***				−0.002	
GeoSES Poverty			−0.842***				−2.341***				−0.012***	
GeoSES Deprivation			−0.304***				−0.249				0.002	
GeoSES Wealth			0.307				−4.576*				−0.105***	
GeoSES Income			−0.001				0.001				0.00002	
GeoSES Segregation			−18.728				−130.314***				−0.966**	
Household Per Capita Income				0.033***				0.086***				0.0002**
Gini Index				−27.756***				−83.147***				−1.378***
Illiterate Rate				−0.265*				−1.899***				−0.015***
Absence of Water and Sanitation				−0.432***				−1.126***				−0.008***
BFP Recipients				−0.160**				−0.430**				0.001
Child Mortality Rate				−0.058				−0.059				−0.001
Constant	98.246***	−60.303***	131.274***	75.987***	191.082***	−273.520***	263.230***	136.169***	3.082***	1.691***	3.259***	3.438***
Marginal R^2 [2]	0.120	0.154	0.128	0.169	0.087	0.103	0.085	0.134	0.043	0.042	0.053	0.099
Conditional R^2	0.291	0.336	0.289	0.366	0.484	0.519	0.456	0.544	0.228	0.230	0.230	0.310

Notes:
[1] *p<0.1; **p<0.05; ***p<0.01 .
[2] Based only on socioeconomic variables.
[3] All adjusted models included Crowding Rate, Demographic Density Rate, State Capitals and Travel Time to State Capital as confounders.

Discussion

We evaluated the importance of municipal socioeconomic factors as a determinant of COVID-19-related mortality rate in Brazil in 2020. More than that, we compared different definitions of mortality rate, where the first was the crude mortality rate, the second was the sex- and age-standardized mortality rate and the third was the sex- and age-standardized mortality rate weighted by the time of the pandemic. To our knowledge, this is the first study to compute and compare diverse COVID-19-related mortality rates at the municipal level in Brazil. In fact, we have not found any other study, in Brazil or in any other country, that has compared results obtained from different definitions, adjusted or not, of mortality rates in regional groupings. The use and comparison of these distinct definitions of mortality rate brought important evidence. When we excluded the effect of demographic structure and pandemic timing from the calculation of mortality rates, the contribution of socioeconomic factors to explain differences in COVID-19-related mortality rates became insignificant. The correlation of the elderly population (a population at very high risk of COVID-19-related death) to the total population and socioeconomic factors were partially responsible for the R^2 found in the crude mortality rate models, and probably not their intrinsic ability to change the population's exposure to infection. Furthermore, the progressive spread of the SARS-CoV-2 virus happened at different speeds and municipalities were at different stages during the pandemic. Hence, weighting by the pandemic time experienced by each municipality partially explains the R^2, since usually the state capitals were the first to be affected by the COVID-19 pandemic in Brazil. Roubaud et al. (2020), the only study that used the entire national data and also included socioeconomic factors under consideration, confirmed our results considering the R^2 coefficient [26].

Our first finding, and the answer to the question indicated in the title, was that socioeconomic factors explained very little of the differences in the municipality-level mortality rate observed in the COVID-19 pandemic in Brazil in 2020. Despite the low ability of socioeconomic factors to explain the mortality rate, we used the model parameters to analyze the influence of each dimension of the socioeconomic status on the COVID-19-related mortality rate in Brazilian municipalities in 2020. We obtained strong evidence pointing out that municipalities with higher income (SVI Labor and

Income, HDI Income and Household Per Capita Income), better education (HDI Education and Illiterate Rate), and urban infrastructure (SVI Urban Infrastructure and Absence of Water and Sanitation) had higher COVID-19-related mortality rates on average when compared to municipalities on average poorer and lacking these resources. It is crucial to highlight that this result is independent of Crowding or Density Rate, healthcare coverage (Physician Rate and ICU Hospital Beds Rate), distance to state capital (Travel Time), urban infrastructure, poverty, income inequality or the distance among municipalities (Haversine Var-Cov matrix), and is repeated among close SES approaches (SVI, HDI and GeoSES). Similar result for income, but not for education, was found by Roubaud et al. (2020) also in a study grouped by Brazilian municipalities [26]. At this point it is interesting to remember that SARS-CoV-2 infections first occurred in capital cities, characterized by higher per capita income, more unequal income distribution, and more pronounced social disparities compared to non-capital municipalities. The virus has progressively interiorized, possibly via the dense urban network that connects capital cities to other municipalities. Consequently, the lack of urban infrastructure, which also includes mobility and transportation structure, influenced the delayed spread of SARS-CoV-2 to small, towns with less urban infrastructure and also lower per capita income. Consistent with this narrative, we also found evidence that more unequal municipalities had fewer COVID-19-related deaths on average.

Undoubtedly, social and economic factors, as already pointed out in several studies, had a relationship with COVID-19-related deaths in Brazil in 2020 [6, 17–21]. Lower per capita income and education, black/brown skin color, household conditions leading to crowding, informal occupation, and specific economic activities were found to be risk factors for COVID-19-related infection and death. However, it is important to note that these studies were based on intra-municipal data, i.e., either individual or grouped by neighborhoods. Our results show that it is not possible to extrapolate these results to municipal aggregates, nor the other way around. Certainly, extrapolation of our results, aggregated by municipalities, to individuals would have a large ecological bias or imply an ecological fallacy. High-income municipalities generally had higher mortality rates due to COVID-19, but it is not possible to say that the wealthier individuals were the most affected and died the most. There is the possibility that the poorer population of

the wealthier municipalities, perhaps less able to adhere to social distancing policies, and therefore at higher presumed risk to infection, died the most in these municipalities. This seems to be the case when we analyze the association between poverty (GeoSES Poverty) and racial segregation (GeoSES Segregation) with mortality rate. Our results show that the higher the deprived population and the more racially polarized the municipality was, the higher the mortality rate was also. Racial inequality and poverty were significant even when considered together with education and income, and their effects remained even when health infrastructure was included in the model. Roubaud et al. (2020) also found that racial minorities, such as African Americans, were more affected even when controlling for income effects on the indicators [26]. In fact, this result was not only reported for Brazil [22, 36]. Roubaud et al. (2020) also found that the greater presence of communities concentrating deprived populations and African Americans, *favelas* or subnormal clusters, was associated with higher SMRT [26]. Although not our primary goal, we can say that the effect of racial inequality on COVID-19-related mortality rate across municipalities was not driven by income, urban condition, education, or health coverage because we used these as controls in the models. The causal mechanism may be some factor that was not considered in our statistical models, and further work would be needed to elucidate this. On the other hand, as for the relationship between pre-existing health condition, embodied in HDI Health, and mortality rate, the results suggested that favorable pre-existing health condition can reduce the probability of death in the municipalities, and that the other way around, poor pre-existing health condition, would be therefore a risk factor to increase COVID-19-related death susceptibility.

The level of analysis is a classic issue in epidemiology. Aggregation into municipalities, an ecological approach, poses the dilemma of using equal or proportional weights to demographics when evaluating associations in models. This problem is not solved solely by using mortality rates, although this implies an important demographic correction. In this study we chose to consider equal weights for the different municipalities because, from a legal point of view, regardless of their population or geographical size, each unit has the same status vis-à-vis the law and their rights. Among COVID-19 studies, we found evidence that the impact of SES on COVID-19-related mortality may vary across levels of aggregation. Knittel e Ozaltun

(2020) found, for example, differences across states and counties in the significance of race and mobility [37].

It is also interesting to note that the narrative of the pandemic at the municipal level in Brazil differs from others observed in high-income countries, where, the impact of the pandemic on mortality was greater in poorer municipalities [13]. The particular social condition of Brazil, the very unequal distribution of wealth and opportunities among different regions, and the concentration of economic activity circuits in the capital cities, may partly explain these differences.

Since our results revealed that socioeconomic determinants did little to explain the increase in COVID-19-related death rates in Brazil in 2020, the question remains as to what these determinants might be. We speculate that a possible determinant would be the lack of federal coordination of public policies agreed with states and municipalities, such as the delimitation of sanitary barriers to reduce the inter-municipal movement of people by public and private transport, or even greater sanitary control of the network that connects municipalities by means of transport, services, and commerce. Another possible factor would be the level of economic development of the municipalities and, consequently, their economic structures. These structures can condition the sector of activity composition and the employment relationship (formal/informal jobs), influencing the worker's ability to adhere to social distancing policies and reduce their exposure to SARS-CoV-2. Thus, a structural economic precondition may exist, which would pose inequalities among municipalities and influence the epidemic burden and mortality.

The robustness of the estimates was a constant concern in this study. To deal with this issue, we took several precautions. First, the mortality rate was standardized by sex and age and weighted by the time of the pandemic. Second, we used the Haversine variance-covariance matrix in all regressions to capture the similarities of nearby municipalities in the significance of the parameters. Third, all models used Crowding Rate and Demographic Density Rate to deal with the natural causes of virus spread due to urban organization, and in addition, Travel Time to State Capital to control for the ease of access of its residents to major (capital) cities. Fourth, additional models were fitted for Hospital Bed Rate, ICU Hospital Bed Rate, Physician Rate, and Nurse Practitioner Rate to control for the possibility of differences in death among counties

due to limited health service coverage (Suppl Material). These additional controls for health service coverage did not change the significance of the R^2 coefficient of the SES and were dropped from further analysis. Sixth, we attempted to work with socioeconomic variables categorized by deciles (first, tenth, and second to ninth) to observe some difference in the coefficients when compared to the numerical possibility. Again, no substantial discrepancies were found, showing the robustness of the results. Our concern apparently paid off. We found great robustness in our results. We used different composite indicators aiming to represent similar socioeconomic dimensions. In this way, different specifications were used to verify the sensitivity of the socioeconomic dimensions to changes in the composition of the indicators. In our results, both the significance and the sign related to the analyzed socioeconomic dimensions change little when we change the indicators selected to represent them.

There are significant limitations to this study. These can be separated into: (i) Lack of available information for some questions in national/municipal perspective; (ii) Specificities of the COVID-19 epidemic data used; and, (iii) Lack of approach to infer causality. Concerning (i), we did not include in the models, meteorological conditions such as temperature, humidity, and rainfall, which have been mentioned in previous studies as a natural cause to influence the spread of SARS-CoV-2 infection [28, 32, 33, 38]. There is a lack of information available on this subject at the national/municipal level in Brazil. However, we included the state of belonging of each municipality as a random effect in the models to control for, among others, these differences in regional weather conditions. Another limitation is the availability of only outdated socioeconomic information, belonging to the last Brazilian census, in 2010. All socioeconomic index groups, HDI, SVI, GeoSES, and those selected from the census itself used the same primary information from the 2010 census in their definitions. Concerning (ii) only hospital deaths were considered and with this COVID-19 related death information is absent for numerous records, not to mention that because of low testing for SARS-CoV-2 infection throughout 2020, especially during the early stages of the pandemic, we may have underestimated or even distorted COVID-19 related mortality rates. We addressed these limitations by including patients with a confirmatory diagnosis of SARS-CoV-2 infection and who had death known to be related to COVID-19. In addition, we use controls in the model estimates to account for

differences in health care resources across counties. Concerning (iii), we chose a cross-sectional study that did not allow us to analyze causal-type relationships between COVID-19-related mortality rate and socioeconomic factors. Previous studies have used the natural experiment design to analyze causality, but only using strong assumptions that are not valid in Brazil, such as the uniformity of social distancing policies across the national territory [13].

Conclusion

Our research concluded that, consistent across all socioeconomic indexes tested, high-income, more educated, and well infrastructure municipalities generally had higher mortality rates. We know, however, that these results cannot be directly reflected in the reality of individual and/or intra-municipal clusters. Although socioeconomic determinants did little to explain the increase in COVID-19-related mortality rates in Brazil in 2020, they did influence the promotion of contamination. In the future, contingency programs should focus on coordinating policies for social isolation and inter-municipal mobility, such as the delimitation of sanitary barriers to reduce the inter-municipal movement of people, or a sanitary control of the network that connects municipalities. More studies are needed to explore other possible determinants of differences in COVID-19-related mortality rates across municipalities, especially social isolation policies.

Supporting information

S1 Fig. Brazilian National Map by Municipalities with Distribution of MortalityRates (a, b and c) and Medians of Mortality Rate by Geographic Locations (d). Brazil, 2020.

S1 Table. Descriptive Analysis of SMRT by Botton and Top Deciles of Attributes ofBrazilian Municipalities. Brazil, 2020.

S2 Table. Adjusted Log-linear Models to Estimate Association Between COVID-19 Mortality Rate (CMR, SMR and SMRT) and Socioeconomic Status inBrazilian Municipalities. Brazil, 2020.

Acknowledgments

This research was funded by Inova-Fiocruz Program: “Programa Fiocruz de Fomento à Inovação - Geração De Conhecimento - Enfrentamento da Pandemia e Pós-pandemia COVID-19 Encomendas Estratégicas” (Grants: VPPCB-005-FIO-20-2-45 and VPPCB-005-FIO-20-2-61).

References

1. Aquino EM, Silveira IH, Pescarini JM, Aquino R, Souza-Filho JAd. Medidas de distanciamento social no controle da pandemia de COVID-19: potenciais impactos e desafios no Brasil. Ciência & Saúde Coletiva. 2020;25:2423–2446. doi:10.1590/1413-81232020256.1.10502020.

2. Barbosa IR, Galvão MHR, Souza TAd, Gomes SM, Medeiros AdA, Lima KCd. Incidência e mortalidade por COVID-19 na população idosa brasileira e sua relação com indicadores contextuais: um estudo ecológico. Revista Brasileira de Geriatria e Gerontologia. 2020;23(1). doi:10.1590/1981-22562020023.200171.

3. Papageorge NW, Zahn MV, Belot M, Van den Broek-Altenburg E, Choi S, Jamison JC, et al. Socio-demographic factors associated with self-protecting behavior during the Covid-19 pandemic. Journal of Population Economics. 2021;34(2):691–738. doi:10.1007/s00148-020-00818-x.

4. Link BG, Phelan J. Social conditions as fundamental causes of disease. Journal of health and social behavior. 1995; p. 80–94.

5. Clouston SA, Natale G, Link BG. Socioeconomic inequalities in the spread of coronavirus-19 in the United States: A examination of the emergence of social inequalities. Social Science & Medicine. 2021;268:113554. doi:10.1016/j.socscimed.2020.113554.

6. Silva J, Ribeiro-Alves M. Social inequalities and the pandemic of COVID-19: the case of Rio de Janeiro. J Epidemiol Community Health. 2021;doi:10.1136/jech-2020-214724.

7. Yasenov VI. Who can work from home? IZA Discussion Paper. 2020;.

8. Adams-Prassl A, Boneva T, Golin M, Rauh C. Inequality in the impact of the coronavirus shock: Evidence from real time surveys. Journal of Public Economics. 2020;189:104245. doi:10.1016/j.jpubeco.2020.104245.

9. McLaren J. Racial Disparity in COVID-19 Deaths: Seeking Economic Roots with Census data. National Bureau of Economic Research; 2020.

10. Benitez J, Courtemanche C, Yelowitz A. Racial and ethnic disparities in Covid-19: Evidence from six large cities. Journal of Economics, Race, and Policy. 2020;3(4):243–261. doi:10.3386/w27592.

11. Abedi V, Olulana O, Avula V, Chaudhary D, Khan A, Shahjouei S, et al. Racial, economic, and health inequality and COVID-19 infection in the United States. Journal of racial and ethnic health disparities. 2020; p. 1–11. doi:10.1101/2020.04.26.20079756.

12. Prats-Uribe A, Paredes R, Prieto-Alhambra D. Ethnicity, comorbidity, socioeconomic status, and their associations with COVID-19 infection in England: a cohort analysis of UK Biobank data. medRxiv. 2020;.

13. Brandily P, Brébion C, Briole S, Khoury L. A poorly understood disease? The unequal distribution of excess mortality due to COVID-19 across French municipalities. NHH Dept of Economics Discussion Paper. 2020;(15).

14. Brown CS, Ravallion M. Inequality and the coronavirus: Socioeconomic covariates of behavioral responses and viral outcomes across US counties. National Bureau of Economic Research; 2020.

15. Chen JT, Krieger N. Revealing the unequal burden of COVID-19 by income, race/ethnicity, and household crowding: US county versus zip code analyses. Journal of Public Health Management and Practice. 2021;27(1):S43–S56. doi:10.1097/PHH.0000000000001263.

16. Sá F. Socioeconomic determinants of Covid-19 infections and mortality: evidence from England and Wales. CEPR Discussion Paper No DP14781. 2020;.

17. Baqui P, Bica I, Marra V, Ercole A, van Der Schaar M. Ethnic and regional variations in hospital mortality from COVID-19 in Brazil: a cross-sectional observational study. The Lancet Global Health. 2020;8(8):e1018–e1026.

18. De Negri F, Galiezz R, Miranda P, Koeller P, Zucoloto G, Costa J, et al. Socioeconomic factors and the probability of death by Covid-19 in Brazil. Journal of Public Health. 2021;doi:10.1093/pubmed/fdaa279.

19. Hallal PC, Hartwig FP, Horta BL, Silveira MF, Struchiner CJ, Vidaletti LP, et al. SARS-CoV-2 antibody prevalence in Brazil: results from two successive nationwide serological household surveys. The Lancet Global Health. 2020;8(11):e1390–e1398. doi:10.1016/S2214-109X(20)30387-9.

20. Miranda P, Koeller P, Zucoloto G, Machado W, De Negri F. Aspectos socioeconômicos da Covid-19: o que dizem os dados do Município do Rio de Janeiro? Instituto de Pesquisa Econômica Aplicada (Ipea). 2020;.

21. Bermudi PMM, Lorenz C, de Aguiar BS, Failla MA, Barrozo LV, Chiaravalloti-Neto F. Spatiotemporal ecological study of COVID-19 mortality in the city of São Paulo, Brazil: shifting of the high mortality risk from areas with the best to those with the worst socio-economic conditions. Travel medicine and infectious disease. 2021;39:101945. doi:10.1016/j.tmaid.2020.101945.

22. Martines MR, Ferreira RV, Toppa RH, Assuncao L, Desjardins MR, Delmelle EM. Detecting space-time clusters of COVID-19 in Brazil: mortality, inequality, socioeconomic vulnerability, and the relative risk of the disease in Brazilian municipalities. MedRxiv. 2020;doi:10.1101/2020.06.14.20131102.

23. Dagnino R, de Freitas MWD, et al. Casos de covid-19 nos municípios do estado do Amazonas, Brasil. Center for Open Science; 2020.

24. Silveira R, Leal O, Soares P, Cruz L, Modesto I, Batista L, et al. Telemonitoramento da COVID-19 com participação de estudantes de medicina: experiência na coordenação do cuidado em Rio Branco, Acre. APS EM REVISTA. 2020;2(2):151–161. doi:10.14295/aps.v2i2.121.

25. da Silva HHC, Dias MdGS. NARRATIVAS SOBRE A PANDEMIA POR COVID 19 NOS ESTADOS DO AMAZONAS E RORAIMA. Somanlu: Revista de Estudos Amazônicos. 2020;1(1):33–49. doi:10.29327/233099.20.1-3.

26. Roubaud F, Razafindrakoto M, Saboia JLM, dos Reis Castilho M, Pero V. The municipios facing COVID-19 in Brazil: Socioeconomic vulnerabilities, transmisssion mechanisms and public policies. UMR LEDa; 2020.

27. Castro MC, Kim S, Barberia L, Ribeiro AF, Gurzenda S, Ribeiro KB, et al. Spatiotemporal pattern of COVID-19 spread in Brazil. Science. 2021;372(6544):821–826. doi:10.1126/science.abh1558.

28. Baumgartner MT, Lansac-Toha FM, Coelho MTP, Dobrovolski R, Diniz-Filho JAF. Social distancing and movement constraint as the most likely factors for COVID-19 outbreak control in Brazil. medRxiv. 2020;doi:10.1101/2020.05.02.20088013.

29. Baggio JAO, Machado MF, Do Carmo RF, da Costa Armstrong A, Dos Santos AD, De Souza CDF. COVID-19 in Brazil: spatial risk, social vulnerability, human development, clinical manifestations and predictors of mortality–a retrospective study with data from 59 695 individuals. Epidemiology & Infection. 2021;149. doi:10.1017/S0950268821000935.

30. Silva APdSC, Holanda ERd, Abreu PDd, Freitas MVdA. COVID-19 in children in the state of Pernambuco: Spatial analysis of confirmed severe cases and the Human Development Index. Revista da Sociedade Brasileira de Medicina Tropical. 2021;54. doi:10.1590/0037-8682-0782-2020.

31. Galvan D, Effting L, Cremasco H, Adam Conte-Junior C. Can Socioeconomic, Health, and Safety Data Explain the Spread of COVID-19 Outbreak on Brazilian Federative Units? International Journal of Environmental Research and Public Health. 2020;17(23). doi:10.3390/ijerph17238921.

32. Rodrigues W, Prata DN, Camargo W. Regional determinants of the expansion of COVID-19 in Brazil. MedRxiv. 2020;doi:10.1101/2020.04.13.20063925.

33. Martins LD, da Silva I, Batista WV, de Fátima Andrade M, de Freitas ED, Martins JA. How socio-economic and atmospheric variables impact COVID-19 and influenza outbreaks in tropical and subtropical regions of Brazil. Environmental research. 2020;191:110184. doi:10.1016/j.envres.2020.110184.

34. Barrozo LV, Fornaciali M, de André CDS, Morais GAZ, Mansur G, Cabral-Miranda W, et al. GeoSES: A socioeconomic index for health and social research in Brazil. PloS one. 2020;15(4):e0232074. doi:10.1371/journal.pone.0232074.

35. Nakagawa S, Schielzeth H. A general and simple method for obtaining R2 from generalized linear mixed-effects models. Methods in ecology and evolution. 2013;4(2):133–142. doi:10.1111/J.2041-210x.2012.00261.X.

36. Demenech LM, Dumith SdC, Vieira MECD, Neiva-Silva L. Desigualdade econômica e risco de infecção e morte por COVID-19 no Brasil. Revista Brasileira de Epidemiologia. 2020;23:e200095. doi:10.1590/1980-549720200095.

37. Knittel CR, Ozaltun B. What does and does not correlate with COVID-19 death rates. National Bureau of Economic Research; 2020. Available from: `https://hdl.handle.net/1721.1/127217`.

38. Auler A, Cássaro F, Da Silva V, Pires L. Evidence that high temperatures and intermediate relative humidity might favor the spread of COVID-19 in tropical climate: A case study for the most affected Brazilian cities. Science of The Total Environment. 2020;729:139090. doi:10.1016/j.scitotenv.2020.139090.

Supporting information

Suppl Table 1. Robustness Check of Adjusted Log-linear Models to Estimate Association Between COVID-19 Mortality Rate (CMR, SMR and SMRT) and Socioeconomic Status in Brazilian Municipalities. Brazil, 2020.

	Dependent variable:											
	CMR				SMR				SMRT			
	(1)	(2)	(3)	(4)	(5)	(6)	(7)	(8)	(9)	(10)	(11)	(12)
SVI Human Capital	−24.237**				−21.681				−0.204			
SVI Labor and Income	−57.826***				−253.282***				−1.133***			
SVI Urban Infrastructure	−8.120*				−6.005				−0.181**			
HDI Education		31.375**				90.157***				0.769***		
HDI Health		−14.946				−58.910				−0.917*		
HDI Income		183.136***				562.364***				2.460***		
GeoSES Education			−0.404***				−1.178***				−0.004	
GeoSES Poverty			−0.865***				−2.368***				−0.012***	
GeoSES Deprivation			−0.343***				−0.326				0.001	
GeoSES Wealth			0.309				−4.154*				−0.092***	
GeoSES Income			−0.001				0.001				0.00003	
GeoSES Segregation			−19.553				−131.645***				−0.925**	
Household Per Capita Income				0.034***				0.089***				0.0003***
Gini Index				−31.498***				−81.751***				−1.168***
Illiterate Rate				−0.240				−1.871***				−0.016***
Absence of Water and Sanitation				−0.441***				−1.148***				−0.008***
BFP Recipients				−0.123				−0.382**				0.001
Child Mortality Rate				−0.043				−0.027				−0.001
Crowding Rate	0.171*	0.295***	0.348***	0.247***	2.570***	3.035***	3.160***	2.914***	0.008***	0.009***	0.010***	0.010***
Density Rate	0.065***	0.051***	0.052***	0.062***	0.199***	0.166***	0.169***	0.188***	0.001***	0.001***	0.001***	0.001***
Travel Time	−0.005	−0.006	−0.005	−0.005	0.001	−0.006	−0.001	0.002	0.0001*	0.0001	0.0001*	0.0002**
State Capital	28.851***	20.356***	27.627***	25.606***	72.746***	45.784***	69.190***	70.116***	0.246**	0.103	0.278**	0.265**
ICU Hospital Beds Rate	−1.514***	−1.844***	−1.786***	−1.620***	−3.338***	−4.515***	−4.151***	−3.976***	−0.039***	−0.044***	−0.040***	−0.040***
Physician Rate	3.825***	1.756*	3.132***	4.697***	5.922***	0.709	5.262**	7.558***	−0.018	−0.039**	0.002	0.002
Constant	97.225***	−62.169***	136.207***	75.571***	193.209***	−288.954***	277.247***	135.709***	3.244***	1.387***	3.474***	3.419***

Notes:
[1] *p<0.1; **p<0.05; ***p<0.01 .

Suppl Table 2. Description of the dimensions of the three socioeconomic status approuches, SVI, HDI and GeoSES.

Index	Composition of variables
**(a) SVI**	
SVI Urban Infrastructure	% of people living in households with an inadequate water supply and sewage system; % of the population living in urban households without garbage collection service; and % of people living in households with per capita income below half minimum wage (2010) who spend more than one hour to get to work
SVI Human Capital	Mortality up to 1 year of age; % of children aged 0 to 5 years who do not attend school; % of people aged 6 to 14 years who do not attend school; % of women aged 10 to 17 years who had children; % of mothers who are heads of household, without complete fundamental and with a child under 15 years of age; Illiteracy rate of the population aged 15 years or older; % of children who live in households in which none of the residents has complete fundamental education; % of people aged 15 to 24 years who do not study, do not work and have per capita household income equal to or less than half minimum wage (from 2010).
SVI Labor and Income	Proportion of people with per capita household income equal to or less than half the minimum wage (2010); Unemployment rate of the population 18 years old and older; % of people 18 years old and older without complete basic education and in informal jobs; % of people in households with per capita income below half the minimum wage (2010) and dependent on the elderly; and, the activity rate of people 10 to 14 years old.
**(b) HDI**	
HDI Education	Average schooling (in years)
HDI Health	Life expectancy
HDI Income	GDP per capita

(c) GeoSES

GeoSES Education	% of people for whose kind of the highest completed degree was higher education; % of people for whose kind of the highest completed degree was master; % of people whose kind of the highest completed degree was doctorate; % of people whose level of education is unschooled or incomplete Primary school; % of people whose level of education is complete primary school and incomplete high school; % of people whose level of education is complete high school and incomplete higher education; and, % of people whose level of education is complete
GeoSES Poverty	Resident density per room; % of people in poverty line: whose per capita household income per month is less than or equal to R$ 255.00 or US$144.89 (half the minimum wage in 2010); % of people in the poverty line and race, black, brown or indigenous; % of people who in July 2010 had a regular monthly income from the Bolsa Família Social Program or the Child Labor Eradication Program (PETI); % of people who in July 2010 had a regular monthly income from other social programs or transfers

GeoSES Deprivation	% of homes with uncoated masonry; % of households with general sewerage; % of households with general water distribution network; % of households with garbage collected directly by cleaning service; % of households with electricity from electricity distribution company; % of households with TV; % of households with washing machine; % of households with refrigerator; % of households with washing machine, TV and refrigerator; % of households with cell phones; % of households with computer with internet access; % of households with mobile phone and internet computer; % of households with motorcycle for private use; % of households with private car; % of households with adequate housing; % of households with access to sewerage, water supply, garbage collection, electricity and adequate housing; % of households without motorcycles or cars ownerships for private use; % of households with only motorcycles ownership for private use; % of households with only private car ownership
GeoSES Wealth	% of rented households with a rental value of R$1,000.00 (US$ 568.20) or more; %age of households with 4 or more bathrooms; %age of people aged 65 years and over with a monthly income equal to or above R$ 5,100.00 (US$ 2,897.72 or 10 Brazilian minimum wages).
GeoSES Income	Monthly household income in 2010 (R$)

GeoSES Segregation	(number of people with income above R$ 5,400.00 - number of people with income below R$ 1,000.00)/number of respondents; (Number of persons with completed higher education - Number of persons without education and incomplete elementary school)/Total respondents; (number of whites with income over R$ 5,400.00 - number of blacks with income equal to or less than R$ 1,000.00)/Total number of people who answered both questions; (number of whites with income over R$ 5,400.00 - number of black + brown + indigenous with income equal to or less than R$ 1,000.00)/Total number of people who answered both questions; and,(number of whites with income over R$ 5,400.00 - number of whites with income equal to or less than R$ 1,000.00)/total number of people who answered both questions
**(d) Other Indexes**	
Household Per Capita Income	Average monthly family income per capita in 2010
Gini Index	Gini Index of household income
Illiterate Rate	% of illiteracy in the population fifteen years old or older in 2010
Absence of Water and Sanitation	% of resident people without access to water and sewerage
BFP Recipients	% of population benefited by the Bolsa Família Program (BFP)
Child Mortality Rate	Children under 1 year old per 1.000 live births

Figure 1

Click here to access/download;Figure;Figure1.tif

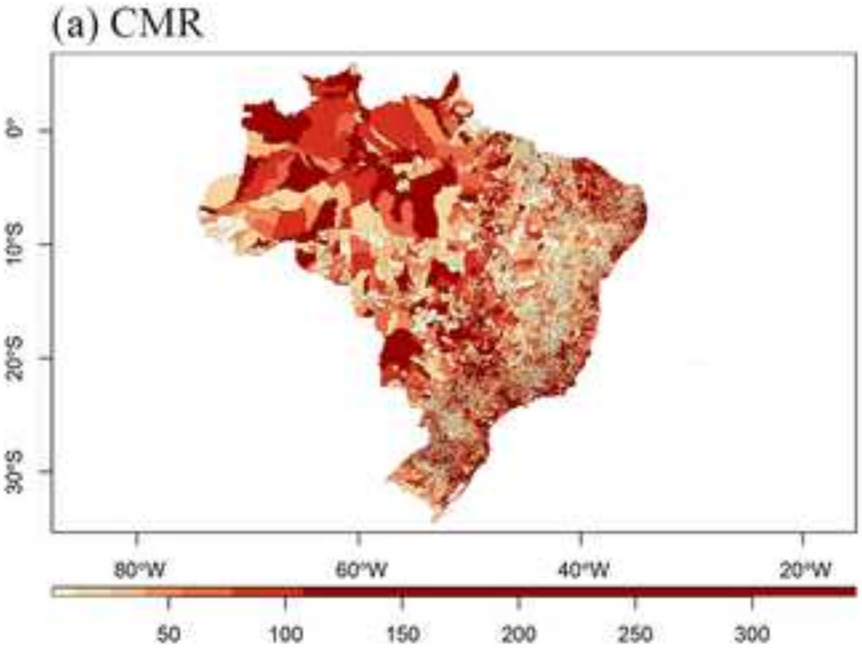

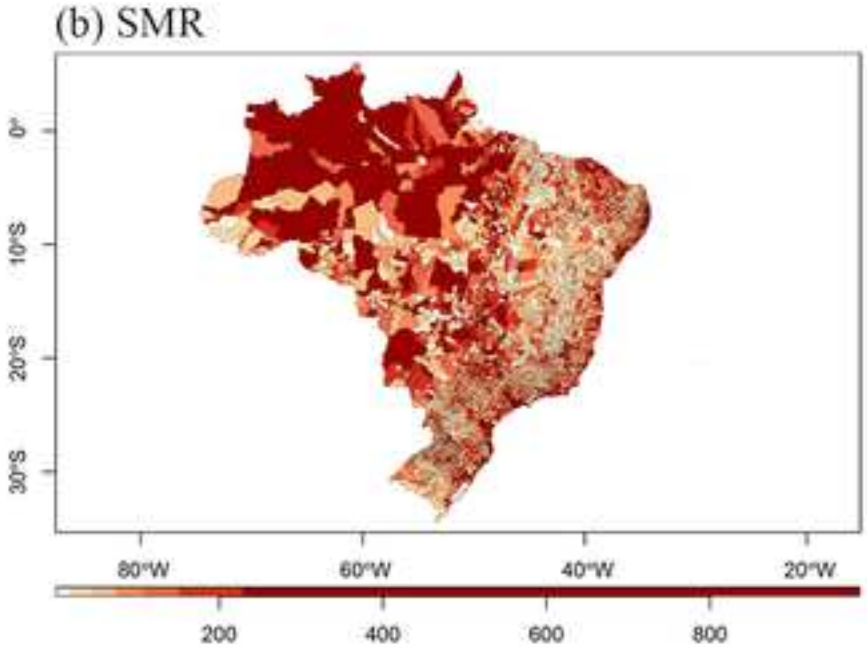

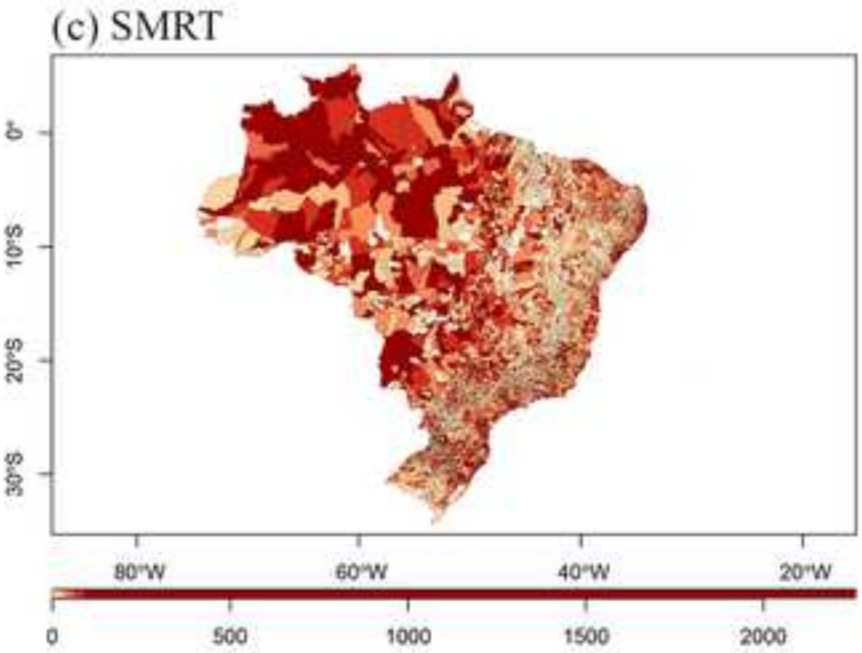

(d) Mortality rate medians

		CMR	SMR	SMRT
Macro-regions	Central-West	64.53	141.41	23.67
	Northeast	45.25	91.74	13.84
	North	65.2	180.58	25.44
	Southeast	56.72	100.61	15.41
	South	55.09	93.43	17.22
Capital	Central-West	151.78	339.27	35.26
	Northeast	144.92	357.34	36.22
	North	162.5	447.57	47.28
	Southeast	165.32	303.05	30.97
	South	133.91	210.5	21.12
	Overall	144.92	361.93	37.1
Non-Capital	Central-West	63.49	141.25	23.58
	Northeast	44.97	91.1	13.76
	North	64.19	177.78	24.66
	Southeast	56.64	100.32	15.39
	South	54.95	93.31	17.18
	Overall	53.32	102.9	16.27
