# Supplementary material for: Were the socio-economic determinants of municipalities relevant to the increment of COVID-19 related deaths in Brazil in 2020?
Source: PLoS One. 2022 Apr 28;17(4):e0266109. doi: 10.1371/journal.pone.0266109 (PMC9049518; doi:10.1371/journal.pone.0266109)
Supplement: Attachment — Submitted filename: Response_to_review.docx [file pone.0266109.s006.docx]

February 10^th^, 2022

Dear Dr. Agricola Odoi

Academic Editor

PLOS ONE

We would like to thank all the reviewers for the manuscript’s helpful comments and suggestions. We thoroughly revised the manuscript and addressed the reviewer’s comments. In addition, we have also taken the recommendations made by the editor in the form of comments in the original document. Changes in the manuscript are indicated in this letter, and in an uploaded new manuscript’s version with changes tracked. A point-by-point response to the reviewers’ comments is provided below. Comments are in regular black font, the author’s answers in standard red font, excerpts from the editions (mostly inclusions) in the manuscript, in *italics*.

Please feel free to contact us with any questions or concerns. We look forward to hearing from you.

Sincerely,

Julio Castro-Alves, PhD

Fundação Oswaldo Cruz, FIOCRUZ

Av. Brasil, 4365 - Manguinhos, ZIP: 21040-360, Rio de Janeiro, RJ, Brazil.

Phone: +55 21 99907-9192

E-mail: julio.castro.alves.lima@gmail.com

REVIEWERS' COMMENTS FOLLOW:

Reviewer #1: The authors investigated whether socioeconomic determinants of municipalities are associated with COVID-19 death rates in Brazil in 2020. Below are my comments:

General comments

1. Area-based indicators of SES are not as robust as the individual-based indicators of SES. While the use of different definitions of mortality rate and SES indicators is useful, given that there are already several studies on this topic using the more accurate individual/person-based indicators of SES, the authors need to clearly discuss why this ecological study is important and the implication of the findings. What novel information does the study add to the existing literature. Why is understanding the relationship between SES and the COVID-19 mortality rate from an ecological perspective important?

A1. We thank the reviewer for this valuable and central question. We agree that many studies discuss the association of SES profile and COVID19 burden in other countries or even in Brazil. However, we are the first study, to our knowledge, to show the impact of different mortality definitions on the association with SES. Most of this literature (ecological studies) used crude mortality rates and found a strong association with SES, while our work did not. Second, we used population data (nationwide dataset), whereas most of the literature chose to select samples from municipalities, states, or compositions. Thus, our conclusions are more robust than those works that used local geographic data. Third, our models included a broad set of variables as confounders to test the robustness of SES, such as the timing of the epidemic in each municipality, the spatial structure of correlation between municipalities, and, as dependent variables, natural causes of virus spread, and health service coverage. Fourth, considering the robustness of the results, we used all SES perspectives available in Brazil to assess our objective, which makes our evidence stronger than most works in the literature. Fifth, at the end of the day, the use of data at the municipal level brought more discussion about distinct determinants for COVID-19 mortality according to the level of analysis, i.e., individual or ecological. We also agree with the reviewer that individual-based studies, in general, are more accurate in bringing evidence to improve public policies. Nonetheless, in the Brazilian case, we do not have the option to develop a nationwide study to examine the association of SES and COVID19 burden at the individual-level. There is no SES information in the surveillance database used to monitor severe acute respiratory infections. Thus, the best alternative was to keep our analysis at the SES most granular level available in Brazil, which is at the municipality level. To incorporate these points into the article, we revised the introduction section and included (Page 4, lines 97 to 109): ”*This work brings some novel and robust information to the existing literature, mainly associated with methodology analysis. So far, we are the first study to our knowledge to address the impact of different mortality definitions on the association of COVID-19 deaths with SES. Most ecological studies used crude mortality rates without examining the implications of this choice on results. In addition, there is a lack of studies investigating the role of socioeconomic inequalities using a robust set of SES indices to explain differences in mortality rates among regions. We examined all available SES data in Brazil. We used a nationwide populational dataset, whereas most literature used samples from municipalities, states, or compositions. The latter can be seen in Brazilian studies but also other countries. Finally, considering statistical modeling aspects, our models included a broad set of variables as confounders to test the robustness of SES. It included the timing of the epidemic in each municipality, the spatial structure of correlation between municipalities, and, as independent variables, natural causes of virus spread, and health services coverage.*”

2. A major limitation of the study that has not been discussed (but was only mentioned) is the use of outdated indicators of SES to assess association with the mortality rate of a disease that occurred almost 10-years later. Do these SES indicators represent the current circumstances? If these SES indicators have not changed in the last 10 years, this should be mentioned or at least discuss possible implications of using outdated indicators on the current findings.

A2. We thank the reviewer for the opportunity to clarify the point of the methodology mentioned above. We only have the population Census in Brazil to provide socioeconomic information about all municipalities. The Census takes place every ten years, the last one was in 2010, and the 2020 one is delayed due to the COVID-19 pandemic. Therefore, we used the best evidence available. Of course, many changes can arise in specific regions or states in a decade, although they are probably not enough to alter the distribution of characteristics among almost 5,000 Brazilian municipalities. We will deepen/extend the discussion of this topic in answer A3.

3. Another limitation that should be included is that use of the 2010 population census to calculate mortality rate would possibly overestimate the rates as the population would have increased between 2010 and 2020.

A3. Good question. We believe it is, in part, the same issue highlighted previously in A2. Outdated information by the municipality can introduce bias in the mortality rate through a change in the pattern of internal migration or population growth. Despite recognizing this possibility, there has been no substantial change in these points in the last ten years capable of affecting the relative distribution of the population in the national territory. To incorporate the issues highlighted in A2 and A3 answers, we expanded the already available discussion section (Page 22, lines 554 to 560) of this topic in the study limitations as follows: “*Another limitation is the availability of only outdated socioeconomic information, belonging to the last Brazilian census, in 2010. All socioeconomic index groups, HDI, SVI, GeoSES, and those selected from the census itself have mostly used the same primary information from the 2010 census in their definitions. It may introduce bias to SES and mortality rate distribution among municipalities. However, there were no significant changes in SES statuses, an internal migration, or populational growth to alter their relative distribution among the almost 5,000 municipalities.*”.

4. The manuscript requires proofreading and editing to correct typographical and grammatical errors to improve the flow and readability, e.g., please use ‘infection’ (or something relevant) instead of ‘contamination’.

A4. We agreed with the reviewer and followed his/her recommendations.

Specific comments

Methods

1. Lines 156-158, are there patients whose COVID-19 diagnosis was determined by imaging only or was this in combination with laboratory tests?

A5. We thank the reviewer for the opportunity to clarify this important aspect of our data. The diagnosis for confirmation of COVID-19 infection followed only one of these options, laboratory, clinical, clinical-epidemiological, or imaging criteria. Our population's most frequent diagnosis confirmation was through laboratory tests, corresponding to more than 90%

2. Line 162, registry should be registrant.

A6. We agreed with the reviewer and followed his/her recommendations.

3. Lines 253-255, please revise the sentence to include the method used

A7. Thanks for alerting us to the editing error. We have replaced the text (Page 9, lines 223-227) “We used the 35) method to obtain the explained variance coefficient (R2) from generalized linear mixed-effects models, partitioned in two definitions. The marginal R2 represents the proportion of variability explained by the fixed effects, and the conditional R2 is the proportion of total variance explained through both fixed and random effects.” by “*We use the so-called "general and simple method" to calculate the two types of coefficients of determination (R^2^) of the adjusted LMMs, marginal and conditional R^2^, where the first represents the proportion of variability explained by the fixed effects and the second represents the proportion of total variance explained through both fixed and random effects [35].*”.

Results

1. Please provide overall descriptive data (number, percentage) on patient/registrant characteristics e.g., mean age, age group, sex, region, capital/non-capital and other available relevant characteristics.

A8. We agreed with the reviewer and followed his/her recommendations. The new Table is available in Supplementary Material (Suppl. Table 3).

2. Please write the results clearly and succinctly for the reader to understand without going back to read up on the different SES indicators and avoid unnecessary repetition, e.g., it is not necessary to use both ‘direct and positive coefficients’ or ‘inverse and negative coefficients’ for the direction of the association between SMRT and SES.

Also, instead of adding the sentences that begin with remember/recall…..you could just write e.g., for lines 371-372: ‘The increase in SVI Income and Labor, which indicates socioeconomic disadvantage, was inversely (P-value < 0.01) associated with increase in SMRT’. Please make the necessary revisions in the results section.

A9. We agreed with the reviewer and followed his/her recommendations. We revised the results section in this new version.

3. Line 274, please include the number of the figure referred to

A10. We agreed with the reviewer and followed his/her recommendations.

4. Figure 1, please include a brief description of the colour gradients for the legend in terms of low/high mortality rate

A11. We agreed with the reviewer and followed his/her recommendations (Page 11, line 248)

5. Table 1, for ease of understanding, can the two columns (1st and 10th) be labelled as low vs high SES or most disadvantaged vs most advantaged?

A12. We edited the title of Table 1 and its column labels to forward the reviewer request. We agreed that the presentation of Table 1 was somewhat confusing. The columns in Table 1 divide low and high municipalities’ mortality rate (SMRT) instead of low and high SES, as suggested by the reviewer.

6. Lines 344-350, this text can be moved to the Methods section under statistical analysis.

A13. We believe that this passage reinforces our focus on presenting the results in the SMRT measure, and so while we understand the reviewer's suggestion, we have chosen to keep it in the results.

7. Line 354-355, note that the outcome was age and sex adjusted with adjustment for the pandemic time, however, these variables were not included in the models as independent variables.

A14. We thank the reviewer for the opportunity to clarify the methodology used in the analyses. The sentence mentioned by the reviewer was “*albeit independently of the age distribution by sex or the pandemic time expected by that municipality.*”. The reviewer is correct. We did not include those variables as independent variables in the models. However, we computed the mortality rate removing the influence of age, sex, and pandemic time. It offers a similar interpretation of the model parameters to the case of including age, sex, and time of pandemic as independent variables, which justify our sentence.

8. Please check the interpretation of the findings on lines 383-386, it does not seem to align with the definitions of the GeoSES Poverty and GeoSES Wealth provided in lines 208-211.

A15. We understand the reviewer's confusion. While GeoSES Wealth is directly proportional to Income, GeoSES Poverty is inversely proportional to poverty. Thus, when both indicators have higher values, they represent that the population has better economic conditions. We have added the information (Page 7, line 177): "*there is an inversely proportional relationship*" in the description of GeoSES Poverty to help the understanding of this indicator, and correct the sentence (Page 15, lines 355 to 358): "These two dimensions of GeoSES agree that the higher the wealth or lower the poverty of a municipality, the higher the average COVID-19-related mortality rate experienced by that municipality in Brazil in 2020." to "*These two dimensions of GeoSES agree that the higher the wealth or lower the poverty of a municipality, the lower the average COVID-19-related mortality rate experienced by that municipality in Brazil in 2020.*".

9. Move the sentence on lines 387-390 to the Discussion section

A16. We agreed with the reviewer. The text “*The relationship between the BFP Recipient population and the SMRT may somehow indicate the efficiency of social security programs, that is, we could not interpret it as increased in poorer municipalities, but rather interpret it as municipalities where the social safety net is more efficient regardless of higher or lower income*” was moved to line 441 to 445, page 18.

10. Lines 391-402, if increasing Gini index, represents disadvantage, and is inversely associated with SMRT, it means that the disadvantaged experienced lower COVID-19-related death rates. Is this right?

A17. Yes, the reviewer is correct in his interpretation. The Gini Index showed a negative relationship with SMRT, and despite this, according to the data studied, municipal socioeconomic indicators were not sufficient to explain the pandemic mortality rate in 2020.

11. Lines 429-433, please revise this sentence so the message is clear.

A18. Thank you for this recommendation. We rewrite the sentence as follows (Page 16, lines 398 to 401): “*While we showed robust correlations between the socioeconomic characteristics of Brazilian municipalities and the COVID-19-related mortality rate in 2020, we also found that the average socioeconomic data of Brazil's municipalities explains very little in the COVID-19-related mortality rate in Brazil.*”

Discussion

1. Lines 500-504, these findings contradict the main study findings of higher SMRT among socioeconomically advantaged communities. The contradictions in these findings should be discussed so the reader can understand why this is the case.

A19. Thank you for this observation. In the mentioned lines, we are talking about segregation and not income. Following the GeoSES Segregation and GeoSES Poverty results, the higher the deprived population municipality was, the higher the mortality rate. And in Brazil, socioeconomically advantaged communities show higher segregation.

2. Lines 514-519, is this speculation or is there evidence for this discussion?

A20. Thank you for the questioning. The sentence mentioned by the reviewer refers to the evidence shown in Table 2, column (10). To elucidate this point, we revised the sentence to include this reference as follows (Page 20, lines 490 to 491): “*(...) poor pre-existing health condition, would be therefore a risk factor to increase COVID-19-related death susceptibility (Table 2, column 10)*”.

3. Lines 558-561, this is the first mention of the sensitivity analyses, please include a sentence about these in the Methods and Results sections.

A21. The sentence mentioned by the reviewer is: *“Fourth, additional models were fitted for Hospital Bed Rate, ICU Hospital Bed Rate, Physician Rate, and Nurse Practitioner Rate to control for the possibility of differences in death among counties due to limited health service coverage (Suppl Material).”*. We opted to keep this analysis in Supplementary Material because it would considerably increase the results section with evidence essentially the same as Table 2. Besides it, the manuscript mentions already the “robustness checks” (Page 9, lines 214 to 217): “*We also did robustness checks using health infrastructure, proxied by ICU Hospital Beds Rate (the number of hospital beds in ICU per million inhabitants in 2015) and Physician Rate (number of physicians per thousand inhabitants in 2015) as controls.*”.

4. Lines 563-566, please provide the results for these analyses as a supplementary for better understanding of the statements.

A22. We agreed with the reviewer and followed his/her recommendations. We also revised this sentence to include the reference of the new table in Supplementary Material (Page 22, lines 538-539): “*Again, no substantial discrepancies were found, showing the robustness of the results* *(Suppl. Table 2)*.”

5. Line 567, delete ‘Our concern apparently paid off’.

A23 .We agreed with the reviewer and followed his/her recommendations.

6. Please include a brief discussion about limitations or caveats associated with ecological studies and how these might affect the current findings.

A24. We thank the reviewer for this important recommendation. In the discussion section (Page 23, lines 568 to 573), we added the text as follows: “*Due to the characteristics of the data available, we carried out an ecological study. In several situations, the use of the data at an individual level can provide more reliable information for developing public health policies. Nevertheless, as the differences between the ecological/individual analysis were extensively addressed in this study, it should be considered more of a point for debate than a limitation.*”

Conclusion

1. Lines 603-605, ‘………………………., they did influence the promotion of contamination’. Please note that the study does not provide data to support this statement. The conclusion should include information that is supported by the study.

A25. We agreed with the reviewer. We rewrote the sentence (Page 23, lines 580 to 584) as follows: “*Our research concluded that, consistent across all socioeconomic indexes tested, high-income, more educated, and well-infrastructure municipalities generally had higher mortality rates, although they did little to explain the increase in COVID-19-related mortality rates in Brazil in 2020. We* *recognize, however, that these results cannot be directly reflected in the reality of individual and/or intra-municipal clusters.*”

Reviewer #2: I have enjoyed reading your article. Please see my comments below.

Please clarify and discuss the following statement:

“At first, elites and high socioeconomic status (SES) communities would become infected because they were not yet prepared to deal with the emerging disease” Could you discuss and describes why this would be the case? Why high SES groups would have higher risk at the beginning of the epidemic? Perhaps they are more mobile, travel more, have higher interaction with other people, etc.

A26. We sincerely appreciate the comments and suggestions of the reviewer. As we mentioned in A1, we revised the introduction to shorten it and also included a discussion of this point as follows (Page 2, lines 53 to 56): “*At first, elites and high socioeconomic status (SES) communities would become infected because they have more opportunities to travel abroad and, consequently, bring the virus to their countries. In addition, the limited knowledge accumulated about the new disease engenders ineffective protocols for dealing with it.*”.

Please discuss more why at the later stages of the COVID-19 epidemic low SES groups had higher infection rates. Perhaps lower access to vaccines, differences in jobs (they must work and cannot stay home to prevent infections, household structure (e.g. more than one generation leaves in a house or more crowded households)

A27. We thank the reviewer for the opportunity to clarify this point. After the first stage, the low SES groups had relatively higher infection rates due to their low ability to mobilize resources to protect their health when there was still no effective protocol to follow. For instance, the exposure to SARS-CoV-2 virus increases in neighborhoods with overcrowded households and poor access to drinking water; due to difference in job quality, such as informal jobs with low wages, few benefits, limited hours of work, and no chance to work remotely; and, susceptibility increases due to chronic comorbidities that follow a pattern motivated by social disparities. We included a new revised text as follows (Page 3, lines 65 to 73): *“As the pandemic progresses,.”*. *exposure to SARS-CoV-2 virus increases in neighborhoods with overcrowded households and poor access to drinking water; or due to differences in job quality, such as informal jobs with low wages, few benefits, limited hours of work, and no chance to work remotely; and susceptibility increases due to chronic comorbidities that follow a pattern motivated by social disparities. So, the presence of a health gradient against racial minorities, black/brown people [9–12] and vulnerable populations [11–14] become even more evident*

Why did you use a log-linear model? You could consider the Poisson or Negative binomial model where the outcome variable would be the number of deceased in a municipality and accounting for the background population as an offset. Alternatively, you could build a Cox Proportional-Hazards Model to investigate the association between the survival time (or the time between exposure and death) of COVID19 patients and SES predictor variables.

A28. We sincerely appreciate the reviewer’s comments and suggestions. There are advantages in using the Poisson, Negative binomial, or Cox Proportional-Hazards models, such as a direct interpretation of risk from the parameters estimated. Due to the lack of SES information in the surveillance database used to monitor severe acute respiratory infections in patients admitted in hospitals, we were not able to use the information at the individual level. For that reason, we ruled out the possibility of using Cox Proportional-Hazards models. On the other hand, aggregating the data to use Poisson or Negative Binomial would demand much more computation (GLMM are known to demand more computational effort than LMM for their optimization) and would make it impossible to simultaneously adjust the ratio of deaths by sex and age, in addition to the demographic one, using the national demographic pyramid as reference. The best alternative was to keep our analysis at the most granulated level where there is available SES information in Brazil, the municipality-level. In this context, we opted to use standardized mortality rates to represent the municipalities, a continuous outcome, which led us to a log-linear model.

EDITOR’S COMMENTS ON TEXT:

You did not investigate these and therefore you should not make concluding statements about it. I recommend you delete it. (lines 33-35)

A29. We thank the reviewer for allowing us to justify the maintenance of the sentence. The first part of the mentioned sentence, “*Excluding the effect of demographic structure and pandemic timing from mortality rates, the contribution of SES to explain differences in COVID-19-related mortality rates among municipalities in Brazil became very low. The impact of SES on COVID-19-related mortality may vary across levels of aggregation.”* was entirely addressed in the result section, and we chose to keep it in the original text. In the second part, “*Urban infrastructure, which includes mobility structures, more complex economic activities, and connections, may have influenced the average municipal death rate.*”, we did not include economic activities and possible connections among municipalities directly in models. This hypothesis was presented in the Discussion section, lines 80 to 82, and should also be kept in the original text.

The introduction is too long and should be shortened.

A30. We agreed with the editor and followed her recommendations. The introduction section was shortened.

Why 2010 data while your COVID-19 data is from 2020. Don't you think this presents problems since you are using SES variables that are 10 years old compared to the disease data?

A31. We hope it is already clarified by the answer given in A2 and A3 when this questioning first appeared.

Considering you are using so many vulnerability variables that are potentially highly correlated with each other, you need to construct and present a conceptual model that shows the perceived relationships between the variables and the outcome and how this guided variable selections and modeling/analysis efforts.

A32. In this study, we use three socio-economic indices: (1) the Social Vulnerability Index (SVI), containing the aggregate index and three other dimensions, i.e., Urban Infrastructure, Human Capital, and Income and Labor; (2) the Human Development Index (HDI), containing the aggregate index and three other dimensions, i.e., health, education, and income; and (3) the Geographic Index of the Socioeconomic Context and Social Studies (GeoSES), containing the aggregate index and seven other dimensions, i.e., education, poverty, wealth, income, racial segregation and deprivation, and services. Besides these, we selected indices/rates among many developed by the Brazilian Institute of Geography Statistics (IBGE, Brazil), composing "something" like a fourth index. Regarding the first three indices mentioned, we know that the composite indexes, usually a linear combination of their dimensions’ values, are therefore highly correlated with their respective dimensions. For this reason, they have been included one-to-one in independent models. As for the various dimensions of a single indicator, we also know that they have been constructed to minimize the correlation between their dimensions, usually using multivariate statistical methods (principal component or factor analysis) to obtain, if not dimensions orthogonal to each other, then as close to it as possible. We did not combine different composite indices from SVI, HDI, or GeoSES into single models, nor did we mix dimensions from any of these indices. As for the socioeconomic indices/markers selected from the IBGE data, we were careful to select those with the lowest covariance among them. Encouraged by this and another question (answered in A36), we will add in the new version of the article (Suppl. Figure 1) the results of this last analysis. We will deepen/extend the discussion of this topic in answer A36.

Please provide details on:

1) How the models were built (ie. how did you select the variables to be included in the final models?). Did you use backward elimination, forward selection , etc?

A33. We thank the editor for this fundamental question of our methodology. Our methodology was designed to evaluate the association of SES and mortality rate in Brazilian municipalities using the regression estimated parameters and R^2^ (coefficient of determination). We used the R^2^ to compare distinct SES indexes. This only works if we keep all regression with the same set of confounders (nested models). Thus, there was no need to use a procedure to select variables among confounders. Besides, we were interested to compute the parameters estimation of all dimensions of each SES index to assess the association of them with the mortality rate. At the end of the day, we were not interested in developing the best model to predict mortality rates. Our objective was to measure the strength of association, its direction, and significance according to distinct SES index and mortality rate definitions.

2) Were confounder assessed and modeled? If yes, how? If no, why not?

A34. We already touched on this subject in answer A33. So, the first answer is YES, we included confounders in all models. For example, in Table 2 we opted to omit the parameters of confounders to save table space, although the footnote contains the description of these. However, in Suppl. Table 1, we added some extra confounders to verify estimation robustness and also showed all parameters estimated, including those of the confounders. The study did not assess the confounder, then the second answer is NO, as was pointed out previously in A33. We used the confounders as described in the subsection “Statistical Modeling” (Page 9, line 211 to 214), as proxies of natural causes of virus spread, such as Demographic Density Rate, and health infrastructure, such as Physician Rate.

3) Were effect modifiers assessed and and modeled? If yes, how? If no, why not?

A35. We thank the editor for this question. Interaction analysis is not part of our methodology described in answer A33.

4) Did you consider the correlations among the explanatory variables? If yes, how and how did you include this knowledge in your modeling effort? If you did not take this into consideration, what is the justification for that decision?

A36. We thank the editor once again for the opportunity to clarify this point. Yes, we considered correlations among the explanatory variables in our analysis. It is important to highlight that socioeconomic variables are naturally correlated among themselves since, for instance, income, education, lifestyle, and access to health services, are usually concomitantly determined over life. Below is a graph with a correlation matrix containing all explanatory variables used in the models. The graph shows that the SES definitions have a high correlation with each other. We used each definition of SES separately in models to deal with it. Contrarily, the confounders generally have a low correlation with different variables, except for “Crowding Rate” (CR). So, we did not need a particular strategy to deal with correlations. Anyway, we included the figure below in Supplementary Material as Figure 1 and also inserted this sentence (Page 9, lines 210 to 211): “*Each of these groups were implemented separately in models to avoid highly correlation (see Suppl Figure 1)*.”

You need to provide confidence intervals for all estimates as well as their exact p-values. (Table 2)

A37. We agreed with the editor and followed her recommendations.

Thank you for considering my comments.
